# D19S Mutation of the Cationic, Cysteine-Rich Protein PAF: Novel Insights into Its Structural Dynamics, Thermal Unfolding and Antifungal Function

**DOI:** 10.1371/journal.pone.0169920

**Published:** 2017-01-10

**Authors:** Christoph Sonderegger, Ádám Fizil, Laura Burtscher, Dorottya Hajdu, Alberto Muñoz, Zoltán Gáspári, Nick D. Read, Gyula Batta, Florentine Marx

**Affiliations:** 1 Division of Molecular Biology, Biocenter, Medical University of Innsbruck, Innsbruck, Austria; 2 Department of Organic Chemistry, Faculty of Science and Technology, University of Debrecen, Debrecen, Hungary; 3 Manchester Fungal Infection Group, Division of Infection, Immunity and Respiratory Medicine, University of Manchester, Manchester, United Kingdom; 4 Faculty of Information Technology and Bionics, Pázmány Péter Catholic University, Budapest, Hungary; Russian Academy of Medical Sciences, RUSSIAN FEDERATION

## Abstract

The cysteine-rich, cationic, antifungal protein PAF is abundantly secreted into the culture supernatant of the filamentous Ascomycete *Penicillium chrysogenum*. The five β-strands of PAF form a compact β-barrel that is stabilized by three disulphide bonds. The folding of PAF allows the formation of four surface-exposed loops and distinct charged motifs on the protein surface that might regulate the interaction of PAF with the sensitive target fungus. The growth inhibitory activity of this highly stable protein against opportunistic fungal pathogens provides great potential in antifungal drug research. To understand its mode of action, we started to investigate the surface-exposed loops of PAF and replaced one aspartic acid at position 19 in loop 2 that is potentially involved in PAF active or binding site, with a serine (Asp19 to Ser19). We analysed the overall effects, such as unfolding, electrostatic changes, sporadic conformers and antifungal activity when substituting this specific amino acid to the fairly indifferent amino acid serine. Structural analyses revealed that the overall 3D solution structure is virtually identical with that of PAF. However, PAF^D19S^ showed slightly increased dynamics and significant differences in the surface charge distribution. Thermal unfolding identified PAF^D19S^ to be rather a two-state folder in contrast to the three-state folder PAF. Functional comparison of PAF^D19S^ and PAF revealed that the exchange at residue 19 caused a dramatic loss of antifungal activity: the binding and internalization of PAF^D19S^ by target cells was reduced and the protein failed to trigger an intracellular Ca^2+^ response, all of which are closely linked to the antifungal toxicity of PAF. We conclude that the negatively charged residue Asp19 in loop 2 is essential for full function of the cationic protein PAF.

## Introduction

Antimicrobial proteins (AMPs) are gaining increased attention as promising new therapeutics to prevent and/or treat microbial infections. The mortality resulting from fungal diseases, particularly in immunocompromised people, has been grossly under appreciated [1]. So far, only few antifungal drugs are licensed to treat deadly diseases in humans and prevent fungal infections in animals and crops [2,3], and resistance against these drugs is increasing. There is thus a great urgency to develop new antifungal drugs with novel targets [1].

**Fig 1:**
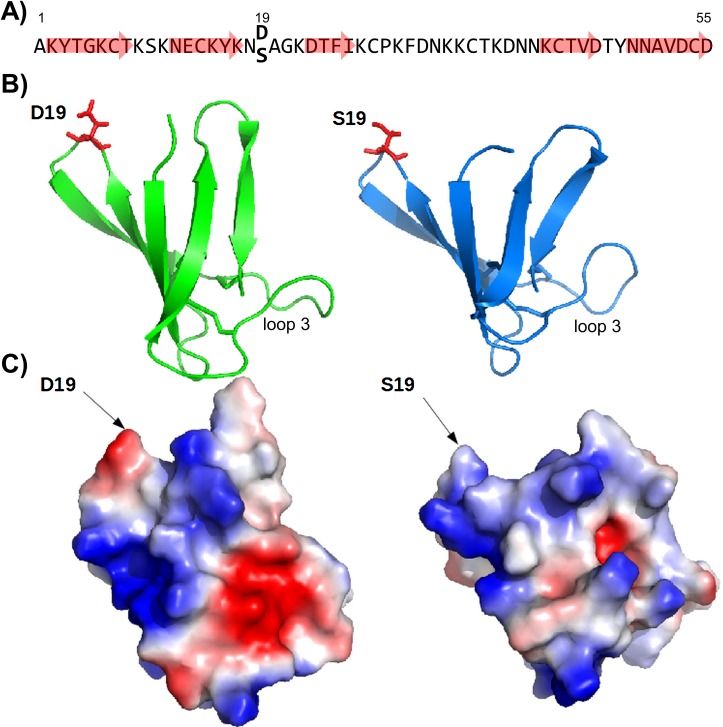
The structural backbone and surface charge of PAF and PAF^D19S^. (***A***) Amino acid sequence of mature PAF and PAF^D19S^ showing the β-strands (red arrows) and the site of amino acid exchange. (***B***) Backbone of the structural ensemble of PAF (left) and PAF^D19S^ (right). Arrows indicate the β-strands that are connected by loops, the Asp/Ser19 exchange is highlighted with red "sticks", respectively. (***C***) Surface representation of PAF (left) and PAF^D19S^ (right) coloured according to electrostatic potential calculated in vacuum (blue: electropositive; red: electronegative). The position of amino acid exchange is indicated by an arrow.

Promising candidates for the development of new therapeutic compounds are small, cationic and cysteine-rich proteins that show potent antifungal activity and are secreted by filamentous Ascomycetes. The knowledge about the structure-function relation of AMPs is an indispensable prerequisite for the exploitation of these molecules in the pharmaceutical industry. One of the best-studied bio-molecules in structure and function is the antifungal protein PAF from the β-lactam producer *Penicillium chrysogenum* [4]. It is a prepro-protein which is processed before secretion and the mature PAF consists of 55 amino acids (Fig 1A) [4]. It specifically inhibits the growth of opportunistic human- and plant-pathogens, such as *Aspergillus fumigatus* and *Botrytis cinerea*, but is inactive against mammalian cells both *in vitro* and *in vivo* [5,6]. In the course of our intensive studies to understand the mechanistic action of PAF, we have investigated its solution structure in great detail [7,8]. PAF exhibits a β-sheet fold that is stabilized by three disulphide bonds: it comprises five β-strands forming two orthogonally-packed β-sheets, which share a common interface. The β-strands are connected by four solvent exposed loops which show increased mobility and structural heterogeneity (Fig 1B) [7–9]. These features point towards an important role of the loop regions in possible protein-host interactions and PAF toxicity [8]. Interestingly, we found in the PAF loop regions 2 and 3 a recurring asparagine-aspartate or aspartate-asparagine sequence preceding or following a lysine residue (Asn18-Asp19 in loop 2, Asp32-Asn33 and Asp39-Asn40 in loop 3) [7].

In the present study, we combined a molecular biology approach with structural analyses and functional tests to gain deeper insight into the structure-function relation of PAF. For this purpose, we examined the role of amino acid Asp19 in the non-conserved loop 2 region in 3D solution structure and antifungal toxicity of PAF by mutating this residue to the fairly indifferent amino acid residue serine (Ser19). Serine was chosen because it is very common in tight protein turns [10], such as loop 2 of PAF where Asp19 is located. Furthermore, it shows a high positive correlation with aspartic acid according to the model of Jonson and Petersen [11], which suggests that a substitution of these amino acid residues exhibits "a high chance of maintaining the thermodynamic stability of the 3D structure". Detailed NMR analyses revealed significant electrostatic surface differences and slight changes in the dynamics at local Ser19 and in the distant loop 3. Thermal unfolding suggested PAF^D19S^ to be rather a two-state folder in contrast to the three-state folder PAF [8]. However, only minor changes in the 3D structure of the mutant protein PAF^D19S^ could be observed when compared with PAF. Functional analyses indicated that the exchange of Asp19 to Ser19 resulted in a severe loss of antifungal activity of the mutated protein. Our data unambiguously prove the importance of this negatively charged Asp19 for the structure and mechanistic function of PAF.

## Materials and Methods

### Strains and growth conditions

Fungal strains used throughout this study are listed in S1 Table. All *P*. *chrysogenum* shaking cultures were inoculated with 10^8^-10^9^ conidia in 200 mL defined minimal medium (MM) and grown for 72 h at 25°C as described previously [12]. Protein isotopic ^15^N-labelling for NMR analysis was performed by replacing the nitrogen source by 0.3% Na^15^NO_3_ (Eurisotop) in MM [7]. *N*. *crassa* was used as PAF-sensitive model organism and cultivated in 5-fold diluted Vogel's medium (0.2 x Vogel's) [13] at 25°C for growth inhibition assays, fluorescence staining experiments and measurements of intracellular Ca^2+^ fluxes. *N*. *crassa* conidia were generated from surface cultures cultivated on Vogel's agar at 37°C for 24 h under continuous light.

### High-yield expression of PAF and PAF^D19S^

An approx. 2080 bp *Pst*I restriction fragment from pSK275*paf*4400 [14] comprising the *paf* gene (420 bp) and approx. 1280 bp of the 5'-UTR and 370 bp of the 3'-UTR was ligated into the *Pst*I site of an empty pSK275 resulting in plasmid pSK275*paf* [14]. For site-directed mutagenesis the preferential codon usage of *P*. *chrysogenum* was taken into account to design two inverse and overlapping oligonucleotides that carried a mismatch sequence coding for the new amino acid replacing the original one (S2 Table). For PCR ligation two overlapping PCR products were amplified, containing the desired mutation (PCR 1: mismatch primer forward and primer M13; PCR 2: mismatch primer reverse and opaf12) and combined in a third PCR reaction using primers T7var and opaf11 (Q5^®^ High-Fidelity DNA Polymerase, NEB). The final PCR product was digested with *Nhe*I/*Not*I and cloned into the *Nhe*I/*Not*I digested pSK275*paf*, replacing the original *paf* sequence. The expression of the mutated *paf* gene was still under the control of the strong *paf* promoter and the expression plasmid was named pSK257*paf*^D19S^. The correct mutation of the *paf* nucleotide sequence was verified using Sanger sequencing (Eurofins/MWG Operon).

In all transformation experiments the *paf* deletion mutant *P*. *chrysogenum* Δ*paf* [14] was used as recipient strain for pSK275*paf* and pSK257*paf*^D19S^ for the production of PAF and PAF^D19S^, respectively. The recipient strain was grown in MM for 48 h at the growth conditions described above and protoplastation and transformation were carried out as described [15,16]. Transformants were selected on MM agar plates supplemented with 0.3-0.6 mg mL^-1^ pyrithiamine hydrobromide (Sigma-Aldrich).

### Protein purification and MS analysis

PAF and PAF^D19S^ were purified from 1 L supernatant of 72 h shaking cultures in MM as described previously [12] with the following changes: After loading of the cleared and ultra-filtered supernatant onto a CM-sepharose column, the proteins were eluted with 0.1-0.3 M NaCl and protein containing fractions were pooled, dialyzed against ultra-pure ddH_2_O, concentrated and filter sterilized. Protein concentrations were determined spectrophotometrically and the purity checked by SDS-PAGE. Proteins were stored at -20°C (in aqueous solution or in lyophilized form for NMR analyses).

The identity of the purified PAF and PAF^D19S^ was proved by the determination of the molecular mass by electro-spray ionization mass spectrometry (ESI-MS) at the Protein Micro-Analysis Facility (Medical University of Innsbruck). In brief, protein samples were dissolved in 50% aqueous methanol containing 0.1% formic acid and injected into a CESI 8000 (SCIEX, USA) coupled to a Q Exactive (Thermo Scientific, 180 nL min^-1^ flow rate). Protein mass determination was performed by deconvolution using the integrated Xcalibur Xtract software (Thermo Scientific).

### NMR measurements, signal assignment and structure calculations

^15^N-PAF^D19S^ NMR sample was prepared by dissolving lyophilized protein in phosphate buffer (10 mM Na_3_PO_4_, 40 mM NaCl, 0.04% NaN_3_, pH = 6.0) to the final concentration of 1.6 mM. NMR experiments for structure determination were carried out at 298 K using AVANCE-II 700 and 500 MHz spectrometers (Bruker). For proton chemical shift referencing DSS (2,2-dimethyl-2-sila-pentane-5-sulfonic acid) was used as external reference and heteronuclear shifts were referenced indirectly from the gyromagnetic ratios for ^15^N and ^13^C. Spectra were processed with TopSpin 3.1 (Bruker) and analysed with CARA 1.8.4 [17]. Sequence specific resonance assignment was obtained from 2D ^1^H–^1^H NOESY (130 ms mixing time), 3D ^15^N HSQC-TOCSY (60 ms) and 3D ^15^N HSQC-NOESY (130 ms) spectra at 298 K. ^13^C-^1^H HSQC spectra were acquired using the natural abundance ^13^C isotopes (80 transients x 640 experiments in the indirect dimension). Sequential resonance assignments were carried out with the identification of NH(*i*)-Hα(*i*-1) distance proximities through the backbone using the combination of 3D TOCSY-HSQC and NOESY-HSQC spectra. Chemical shifts of PAF (BMRB: 19657) were used to support the resonance assignment and structure refinement of PAF^D19S^. For the calculation of PAF^D19S^ structure, data were collected from 2D ^1^H-^1^H NOESY only. After initial calculations with NOE data, structure refinement was performed using C_α_ and C_β_ chemical shift data, and disulphide restraints. Backbone torsion angles were calculated with TALOS+ [18,19]. This procedure had no effect on the overall fold of conformational ensemble, but backbone RMSD values were lower than without torsion angle restraints. Cyana 2.1 algorithm was used in combination with Atnos/Candid procedure for structure calculations [20] and NOE assignment, respectively. Disulphide pattern of PAF^D19S^ was assumed to be identical with that of the wild-type PAF [9], and was given explicitly as covalent bond restraint input to Cyana. Ensembles of 100 structures were calculated and from those, 20 calculated structures were selected according to lowest energy. Structure visualization and analysis was done using MOLMOL [21] and in-house scripts. ^15^N-CEST NMR experiments and temperature dependent experiments were performed as described before [8]. In order to obtain comparable datasets, the same instrumentation was used in the same temperature range (265-343K). Modelling of two-state thermal unfolding was carried out using in-house written MATLAB^®^ scripts as described before [8]. CLEANEX NMR data were fitted against the theoretical function using an in-house written MATLAB^®^ script to yield NH-H_2_O exchange rates as described [22].

### Antifungal activity assays

The growth inhibition assays were carried out in 96-well plates (Nunclon^®^D, Thermo Scientific) as described [7,23]. Briefly, 10^3^-10^4^ *N*. *crassa* conidia were incubated with increasing concentrations of PAF and PAF^D19S^ in liquid medium in a total volume of 200 μL per well. Where appropriate, 0-10 mM CaCl_2_, MgCl_2_ or NaCl were added. The fungal growth was monitored microscopically and by measuring the optical density (OD_620nm_) after 24-48 h of incubation (25°C) with a GENios Plus Microplate Reader equipped with Magellan software (Tecan). The minimal effective concentration (MEC) was defined as concentration that reduced growth by ≥ 90%. The germination efficiency and germ tube length of *N*. *crassa* was determined by incubating 5 x 10^4^ conidia mL^-1^ in liquid medium with 0-32 μM antifungal proteins or 50% ethanol (control) at 25°C for 6 h under continuous stirring. Approx. 100 conidia were analysed for the presence of germ tubes and the tube length was measured by using AxioVision software (Zeiss). Fungistatic and fungicidal effects of PAF and PAF^D19S^ were determined by the method of Muñoz et al. [24]. *N*. *crassa* conidia (10^4^ mL^-1^) were incubated in distilled water with 0-32 μM PAF, PAF^D19S^ or 50% ethanol (control) at 25°C for up to 24 h under continuous stirring. Samples were taken at different time points, diluted and plated on Vogel's agar containing 0.002‰ (w/v) dichloran to slow down colony expansion [25]. The plates were prepared in duplicates and incubated at 37°C for 24-48 h to determine colony numbers. To visualize cell death by pore formation in the cell membrane propidium iodide (PI) staining was performed on 2.5 x 10^5^ conidia mL^-1^ in liquid medium or on conidia germinated on cover slides for 6-30 h at 25°C. The conidia/germlings were exposed to 32 μM PAF and PAF^D19S^ for various times at 25°C and then stained with 0.5 μg mL^-1^ PI for 5 min. All experiments were prepared at least in duplicates and repeated twice and statistical calculations were done with Microsoft Excel. Statistical significance was evaluated using Student’s two-tailed t-test.

### Labelling of PAF and PAF^D19S^ with the fluorophore BODIPY

The proteins were labelled with the green fluorophore BODIPY FL EDA (Life Technologies) as described with minor changes [26]. In brief, protein samples (0.4 mM) were dissolved in 0.1 M MES buffer pH = 4.5. BODIPY was added to a final concentration of 10 mM and subsequently EDAC (Life Technologies) and Sulfo-NHS (Life Technologies) were added to a final concentration of 10 mM and 5 mM, respectively. The reaction mixture was stirred in darkness for 3 h at 25°C, followed by dialysis against ddH_2_O. Protein concentration and degree of labelling were determined spectrophotometrically.

### Microscopy

Microscopic images were generated with a CK40 microscope (Olympus) equipped with an Axiocam MRm camera (Zeiss). PI staining experiments, the uptake and localization studies of fluorescence labelled PAF proteins were analysed with a Zeiss Axioplan fluorescence microscope, equipped with an AxioCam MRc camera (Zeiss, excitation/emission filters 365/420 nm for blue fluorescence, 500/535 nm for green fluorescence, 546/590 or 565/620 nm for red fluorescence). For imaging, special care was taken to apply the same exposure times in each experiment to allow direct comparison of the fluorescence signal intensities between samples. Image editing was done with GIMP (GNU Image Manipulation Program, version 2.8.10).

### Aequorin-based measurement of intracellular Ca^2+^ fluxes in response to PAF and PAF^D19S^

The measurement assays of [Ca^2+^]_c_ concentration were performed as described previously [23]. In brief, conidia of the *N*. *crassa* strain expressing AeqS [27] were suspended in liquid 0.2 x Vogel's media containing 2.5 μM of the co-enzyme coelenterazine (Biosynth AG) and incubated at 25°C for 6 h in the dark. After adding the antifungal proteins, a multimode plate reader (TriStar LB 941, Berthold Technologies) was used to measure bioluminescence at 25°C, over a time course of 60 min, taking measurements of the relative light units (RLUs) per well for 95 cycles (a cycle being the time it takes to measure all wells in the experiment). To convert relative light units (RLUs) into dynamic measurements of micromolar [Ca^2+^]_c_ in populations of conidial germlings during an experiment, an empirically derived equation was used as previously described [28,29].

## Results

### The production of PAF^D19S^

PAF^D19S^ was generated applying PCR-based mutagenesis of the *paf* gene. We took advantage of the *P*. *chrysogenum paf* gene deletion mutant (strain Δ*paf*) [14] to insert a plasmid carrying the mutated *paf* gene. After three rounds of single spore isolation, positive transformed *P*. *chrysogenum* PAF^D19S^ clones were tested for best protein production over a time course of 96 hours before one positive clone was selected for the highest secretion of PAF^D19S^ into the supernatant (data not shown). PAF^D19S^ was purified in a single-step chromatography and eluted from the cation-exchange column with 0.3 M NaCl.

The purity of the protein preparation and the identity of PAF^D19S^ were verified by ESI-MS (S1 Fig). A single peak corresponding to the average mass of 6.215 kDa for PAF^D19S^ was detected. These data correlated with the calculated theoretical mass (web.expasy.org/protparam) of the oxidized protein form indicating the presence of three intra-molecular disulphide bonds and the absence of post-translational modifications, except for cleavage of the pre-pro sequence, as reported also for wild-type PAF (Fig 1A and 1B) [7]. The overall net charge of PAF at pH 7 changed from 4.7 to 5.7 in PAF^D19S^ (web.expasy.org/protparam).

### PAF and PAF^D19S^ exhibit similar solution structures, but differ in their surface charge distribution, dynamics and thermal unfolding properties

To determine the effect of the amino acid exchange on the protein solution structure the protein variant PAF^D19S^ was ^15^N-labelled and analysed by NMR spectroscopy. The chemical shift dispersions of the protein variant compared to the chemical shifts of PAF pointed towards a very similar folded overall structure of both proteins. This was further supported by comparable ^13^C chemical shifts (from natural abundance ^13^C isotopes), which are strong indicators for backbone conformation (S3 Table).

Using conventional NOE distance constraints, the 3D solution structure of PAF^D19S^ was determined (PDB ID: 2nb0, BMRB ID: 25957). With the exception of the exchanged Ser19 residue and its neighbour Ala20, the maximum ^13^C chemical shift difference was lower than 0.2 ppm compared to the PAF resonances (S3 Table). This indicated highly similar structures with only a minor change in backbone conformation around the mutated residue. At Ser19, PAF^D19S^ lost its negative charge and this electrostatic change was further relayed to distant regions of the protein, most remarkable reflected in alterations in the local conformation of the loop between residues 32–41 (Fig 1C and S2 Fig), demonstrating a serious consequence of the mutation.

Analysis of the lysine residues in terms of their backbone and side-chain conformation as well as their closest negatively charged spatial neighbour reveals a remarkable redistribution of the positively charged side-chains at the surface relative to wild-type PAF, also supported by differences in the NOE pattern between the two molecules (S3 Fig). Importantly, the loss of a large negatively charged surface patch is the consequence of the C-terminal Asp55 getting buried by lysine side chains in the D19S mutant.

**Fig 2:**
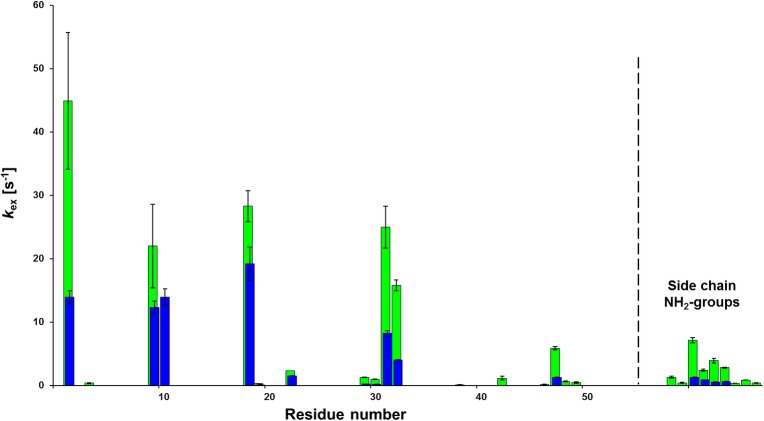
Comparison of NH-water exchange rates measured in CLEANEX NMR experiments. The NH-water exchange rates (s^-1^) are shown for PAF (green) and PAF^D19S^ (blue) at 298 K and pH = 6.0. Note that the very slow exchange rates from residues located in β-strands are not available by this technique, while measurement of fast exchange is inaccurate as shown by the error bars. Some Asn side-chain carboxamides are separately shown (to the right from the dashed line from residue 55: N18, N33, N40, N49, N50).

CLEANEX NMR exchange experiments [22] showed that many of the residues in loop regions gave *k*_ex_,_NH-H2O_ values in the range of 0.1–50 s^-1^. Ser19, as a part of short loop 2, remained solvent accessible (*k*_ex,NH-H2O_ = 19 ± 3 s^-1^, pH = 6) like Asp19 in PAF (*k*_ex,NH-H2O_ = 28 ± 3 s^-1^, pH = 6) (Fig 2 and S4 Fig). However, the exchange rates and therefore the solvent accessibility generally decreased at the solvent exposed loop regions of PAF^D19S^ and at Asn side-chain carboxamides (Fig 2 and S4 Fig). The decrease of the exchange rates in PAF^D19S^ may be a consequence of the increase of the protein net charge (6.06 vs. 5.07) that could potentially strengthen the first hydration shell, diminishing the access to bulk water. We want to note at this point that the very slow exchange rates from residues located in β-strands are not available by this technique.

**Fig 3:**
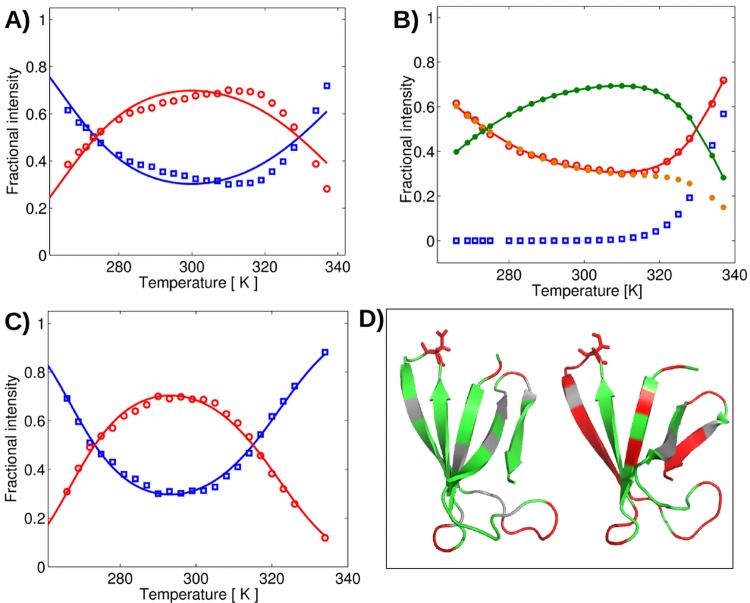
Modelling of thermal unfolding of PAF and PAF^D19S^. (***A-C***) Fitting of a representative residue Ala20 by the two-state (***A*** for PAF, ***C*** for PAF^D19S^), and the three-state unfolding model (***B***) for PAF. Both folded (red ○) and unfolded (blue □) fractions are shown on ***A*** and ***C***. While Ala20 could not be fit below 6% fit error (***A***, 10.01%) in case of PAF, fitting by the three-state model was successfully applied (***B***, fit error = 1.96%, folded: green ●, unfolded 1: orange ●, unfolded 2: blue □, sum of unfolded 1 and 2: red ○). In case of PAF^D19S^ Ala20 is rather a two-state folder (***C***, fit error = 3.37%). The estimated relative error of the experimental 2D peak volumes in the ^15^N-^1^H HSQC spectra must be within 5%, according to repeated experiments. (***D***) Ribbon representation of PAF (left) and PAF^D19S^ (right), displaying the error limits of the thermal unfolding experiment fits. Red: residues that could be fit by the two-state model below 6% error limit. Green: residues that could not be fit below the error limit by the two-state model. Grey: not detected residues (signals could not be integrated because of overlaps). "Sticks" represent the site of the mutation (Asp/Ser19).

According to ^15^N-relaxation data at 298 K and pH = 6, the dynamics was slightly enhanced around residue Ser19. The *S*^2^ order parameter showed a decrease of 0.1 units at Ser19, in contrast to the smooth *S*^2^ function around Asp19 in PAF (S5 Fig) [7]. Similar to PAF [8], weak but unambiguous ^15^N CEST effects were observed around the termini of PAF^D19S^, at residues Tyr3, Thr47, Ala51 and Asp53, displaying the presence of distinct, low populated conformers. These minor conformers (< 1%) exchange slowly (~150/s) with the major conformer and exhibit extreme ^15^N^H^ chemical shift for example in case of Asp53, which was shifted by approx. -11 ppm with respect to the major conformer (S6 Fig). Three more residues in PAF^D19S^ have nearly identical CEST shifts as in PAF (Tyr3: -5 ppm, Thr47: -6 ppm, Ala51: +4.5 ppm), suggesting that the presence of the same sporadic conformer is not influenced by the D19S mutation. Thermal unfolding experiments of PAF^D19S^ showed surprisingly different parameters compared to PAF. In PAF most of the residues could not be fitted using a two-state model (below arbitrary 6% fitting error) with the exception of some non-conserved residues in loop regions, namely Lys2, Ser10, Lys11, Asp19, Asp32, Asn33 and Tyr48 as described before [8]. In the pertinent fitting protocol used for all experiments the fit errors for residues K30 and F31 in PAF were slightly above the arbitrary 6% limit (6.8% and 8.8%, respectively) and as a result, even more residues turned from three-state to two-state folder if based on these formal criteria. In contrast, the fit errors were significantly lower in PAF^D19S^ and the range of "two-state folder" residues increased considerably when applying the same criteria (S7 Fig). All the "two-state folder residues" in PAF remained two-state folders in PAF^D19S^. However, in addition to these regions residues 4, 6, 12, 20, 21, 23, 25–28, 30–33, 47, 48, 51–53 and 55 could be fit by the two-state thermal unfolding model in this PAF mutant (Fig 3). This observation is consistent with a putative scenario in which hidden intermediate states are important contributors to PAF function [8].

### The exchange of aspartic acid 19 to serine severely reduces the antifungal activity of PAF

We next analysed the impact of the amino acid exchange on the toxicity of PAF. The PAF-sensitive test organism *N*. *crassa* was exposed to 2-fold increasing protein concentrations (0-128 μM) and the growth rates of the treated samples were compared with the untreated control. Conidia of *N*. *crassa* have been previously shown to germinate in the presence of inhibitory concentrations of PAF, but their subsequent growth is inhibited [23]. The growth of *N*. *crassa* germlings was reduced by 90% after 30 h of incubation in the presence of 0.06 μM PAF and 32 μM PAF^D19S^. The MEC of the protein variant was significantly higher than the MEC of PAF indicating that PAF^D19S^ severely lost antifungal activity.

Compared to untreated controls no difference in germ tube lengths could be detected in 6 h-old *N*. *crassa* germlings exposed to the MEC of PAF (0.13 μM: 40.5 ± 10.5 μm versus 47.0 ± 3.7 μm in the untreated control). However, when applying 32 μM PAF (250-fold higher than its MEC) the germ tube length reached only 50% of the control (22.6 ± 3.6 μm; *p* = 0.04). In contrast, PAF^D19S^ reduced the germ tube length by 50% of the control at a concentration corresponding to its MEC (32 μM: 25.3 ± 4.1 μm; *p* = 0.06). No germination was observed in ethanol-treated control conidia.

Our results showed that none of the tested proteins inhibited the spore germination in *N*. *crassa* but exclusively reduced germ tube length in a concentration dependent manner. The differences in antifungal activities suggested an altered mechanism of action of PAF^D19S^ compared to that of PAF.

### The antifungal activity is specifically Ca^2+^ ion sensitive

In previous studies we reported that the extracellular ion concentration influences the antifungal activity of PAF [12,23]. To compare the ion sensitivity of PAF and PAF^D19S^, we supplemented the growth medium with increasing concentrations of monovalent (NaCl) and divalent ions (CaCl_2_, MgCl_2_). The test medium (0.2 x Vogel's) *per se* contained 0.03 mM CaCl_2_, 0.16 mM MgCl_2_ and 2.16 mM Na_3_-citrate. No considerable effect on the activity of PAF and PAF^D19S^ could be observed with the addition of 0–10 mM NaCl (Table 1). Instead, the divalent cations CaCl_2_ and MgCl_2_ reduced the toxicity of PAF and PAF^D19S^ in a concentration dependent manner, whereby the PAF-neutralizing effect of Ca^2+^ ions was more prominent than that of Mg^2+^ ions (Table 1). The little growth of *N*. *crassa* at 0.06 μM PAF (1.7 ± 0.5% growth) could be increased to 92.6 ± 3.9% by adding 10 mM CaCl_2_ to the culture medium. The addition of 10 mM MgCl_2_ was less effective than CaCl_2_ and ameliorated growth under PAF challenge only to 54.0 ± 2.0%. The addition of 0.3 mM divalent ions had no growth promoting effect. In contrast, PAF^D19S^ was significantly more ion sensitive than PAF. The addition of only 0.3 mM CaCl_2_ to the culture medium ameliorated the low fungal growth in the presence of 32 μM PAF^D19S^ (2.6 ± 0.6% growth) to 52.1 ± 4.6% growth and 10 mM of this ion further improved growth to reach 94.6 ± 12.1%. The growth of PAF^D19S^-exposed *N*. *crassa* cells could be significantly ameliorated with the addition of 10 mM MgCl_2_ (79.5 ± 2.1%).

**Table 1:** The effect of ion supplementation of the culture medium on the growth-inhibitory activity of PAF and PAF^D19S^ in *N*. *crassa*.

	% Growth (mean ± SE) with^a^						
Protein	no ions	CaCl_2_		MgCl_2_		NaCl	
	0	0.3	10	0.3	10	0.3	10
PAF	1.7 ± 0.5	2.4 ± 1.7	92.6 ± 3.9	2.3 ± 0.9	54.0 ± 2.0	0.8 ± 0.5	4.2 ± 0.8
PAF^D19S^	2.6 ± 0.6	52.1 ± 4.6	94.6 ± 12.1	2.4 ± 0.7	79.5 ± 2.1	2.3 ± 1.2	12.7 ± 4.0

^a^The OD_620_ was measured after 30 h of incubation. The growth of the untreated controls in the absence or presence of ion supplementation was normalized to 100% to evaluate the percent growth (mean ± standard error, SE) of samples exposed to the MEC of PAF (0.06 μM) and PAF^D19S^ (32 μM) without addition (no ions) and after addition of ions to 0.2 x Vogel’s at the indicated concentrations in [mM].

Our results demonstrated that the antifungal activity of PAF was most sensitive to Ca^2+^ ions and this sensitivity was most pronounced in PAF^D19S^. This pointed towards an important role of Asp19 in the Ca^2+^-dependent antifungal mode of action of PAF.

### PAF^D19S^-uptake in *N*. *crassa* is impaired

We could show previously that the toxic activity of PAF is closely linked with its binding to and active internalization by PAF-sensitive fungi [30]. We therefore investigated and compared the uptake and localization of PAF^D19S^ with that of PAF. To this end we labelled both proteins with the green fluorescent dye BODIPY and verified that they had comparable labelling efficiencies and a similar antifungal activity to the unlabelled proteins (data not shown). To visualize the fluorescence signals protein conjugate concentrations of 0.8-32 μM were applied.

**Fig 4:**
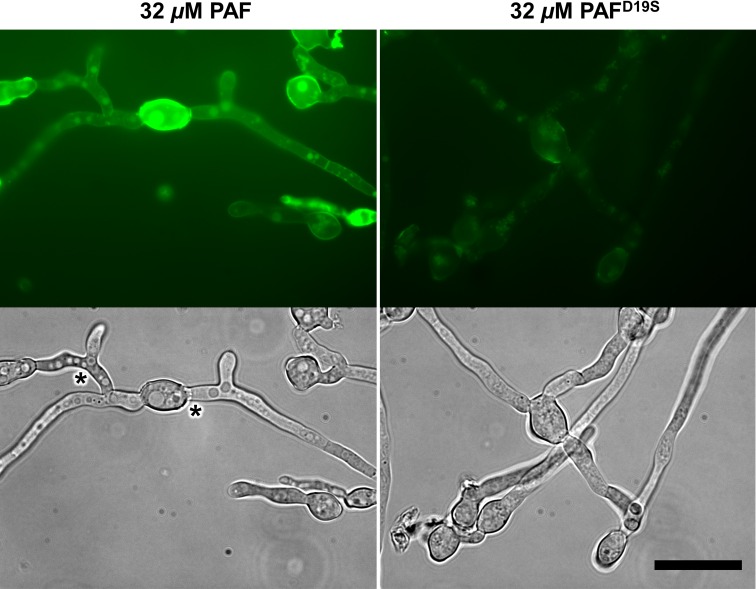
The localization of BODIPY-labelled PAF and PAF^D19S^ in 6 h-old *N*. *crassa* germlings. After the treatment for 1 h with 32 μM BODIPY-PAF the conidial cell wall, vacuoles and the cytoplasm were stained. PAF treated hyphae showed large vacuoles (asterisks, left panels). Only weak signals at the outer layers and in the cells could be observed with BODIPY-PAF^D19S^. Scale bar = 20 μm.

BODIPY-PAF accumulated in the outer regions (cell envelope) of *N*. *crassa* conidia within the first minutes after addition (S8 Fig), and with longer incubation time localized in vacuoles and the cytoplasm (Fig 4 and S9 Fig). Uptake of BODIPY-PAF resulted in the formation of large vacuoles in conidia as well as in hyphae (Fig 4). BODIPY-PAF did not interact with the PAF-insensitive fungus *A*. *terreus* [12] even after 1 h of exposure (S9 Fig), which proved staining specificity and correlated with previous findings [30].

**Fig 5:**
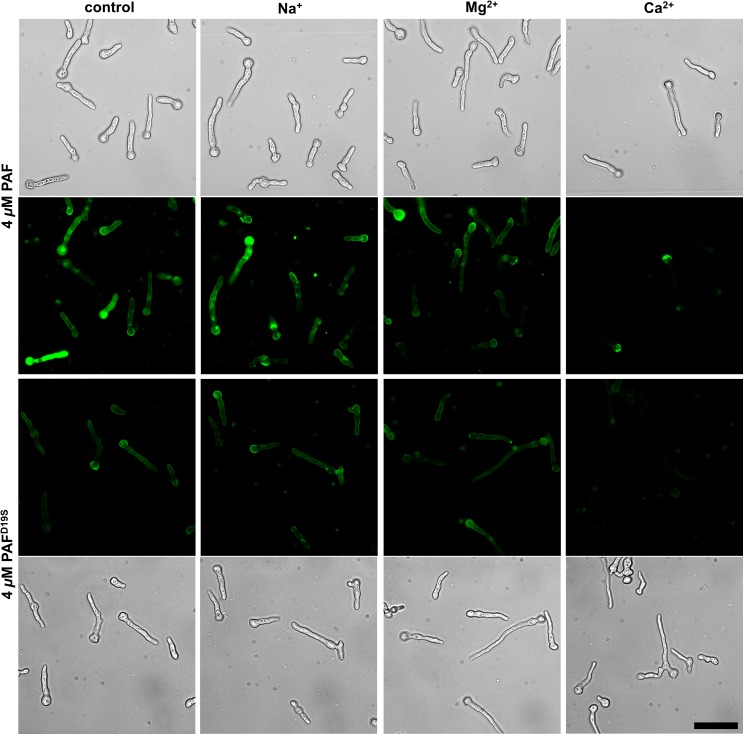
The effect of the addition of ions to the growth medium on binding and uptake of BODIPY-labelled PAF and PAF^D19S^. Conidia of *N*. *crassa* were germinated in the presence of 4 μM BODIPY-PAF or BODIPY-PAF^D19S^ for 6 h in liquid medium supplemented with 1 mM NaCl, MgCl_2_ and CaCl_2_, respectively. In the control the 0.2 x Vogel’s growth medium was without ion supplementation. Scale bar = 30 μm.

We next investigated the localization of BODIPY-PAF^D19S^. The germination of *N*. *crassa* conidia in the presence of 4 μM BODIPY-PAF^D19S^ resulted in very weak fluorescent staining, and this was primarily associated with conidia (Fig 5). When BODIPY-PAF^D19S^ was applied at its MEC (32 μM) on germlings, the fluorescent staining slightly increased and was also more visible in hyphae (Fig 4).

The impaired antifungal activity of PAF^D19S^ was reflected by reduced protein binding and internalization, two events that are indispensable for the full antifungal activity of PAF [30]. Therefore, it is plausible that this weak protein–target interaction may be readily disturbed by the addition of low Ca^2+^ concentrations as observed in growth inhibition assays. To address this latter hypothesis, we performed staining experiments in the presence of increasing ion concentrations in the culture broth. The very low fluorescence signals of BODIPY-PAF^D19S^ in *N*. *crassa* conidia and germlings were slightly diminished with the addition of 1 mM MgCl_2_ and readily disappeared with 1 mM CaCl_2_ supplementation in the medium (Fig 5). At the same Ca^2+^ ion supplementation of the medium (1 mM) the intracellular signals of BODIPY-PAF completely disappeared in the germ tubes but not in the conidia (Fig 5). No effect in the staining intensity and pattern of BODIPY-PAF and BODIPY-PAF^D19S^ had the supplementation with NaCl (Fig 5).

The greater fluorescent labelling of the conidial cell envelope (comprised of the cell wall and plasma membrane) with PAF and PAF^D19S^ suggested that this cell structure exhibits a greater binding affinity to both proteins. Furthermore, our results indicate that Ca^2+^ ions (directly or indirectly) most effectively disturb the interaction of PAF and PAF^D19S^ with the target fungal cells and prevent protein uptake.

### PAF^D19S^ fails to disrupt intracellular Ca^2+^ homeostasis

**Fig 6:**
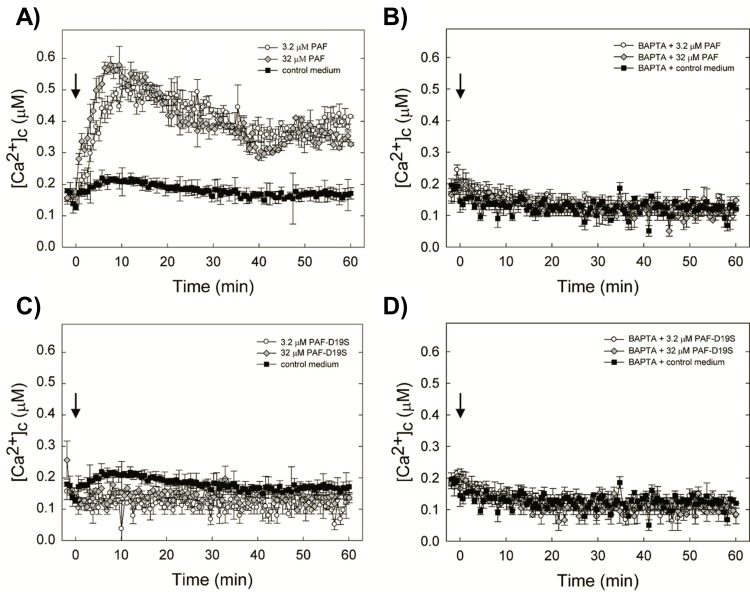
The effect of PAF and PAF^D19S^ on the [Ca^2+^]_c_ resting level of 6 h-old *N*. *crassa-AEQ* germlings. Proteins were added (arrow at time point 0) at a final concentration of 3.2 and 32 μM to the growth medium (***A***, ***C***) and in medium pre-treated with 5 mM BAPTA (***B***, ***D***). Top panels for PAF and bottom panels for PAF^D19S^. Measurements were taken every 40 sec over a period of 60 min. Samples treated with medium without the antifungal protein served as controls. Values represent the mean of three samples ± standard errors.

Another indication for the fungal cell killing activity of PAF is the induction of the perturbation of the intracellular [Ca^2+^]_c_ homeostasis [23,31]. To further characterize the loss of the antifungal activity of PAF^D19S^, we analysed the influence of this PAF variant on [Ca^2+^]_c_ homeostasis. To measure [Ca^2+^]_c_ changes, we used the transgenic *N*. *crassa-AEQ* strain that expresses the gene *aeqS*, coding for the Ca^2+^-sensitive photoprotein aequorin [27,32]. This strain has been previously shown to exhibit the same susceptibility to PAF as the untransformed *N*. *crassa* wild-type strain [23]. As expected, PAF elicited a fast and sustained elevation of [Ca^2+^]_c_, which corresponded well with our previous studies [23,31]. After 60 min of measurement the [Ca^2+^]_c_ maintained a steady level of 0.35-0.45 μM for 3.2 μM and 32 μM PAF (Fig 6A). In contrast, the [Ca^2+^]_c_ in the PAF^D19S^-treated samples remained virtually unchanged at the same level as that of the media-treated control (0.15-0.2 μM Ca^2+^) (Fig 6C). The addition of the extracellular Ca^2+^-chelator BAPTA (5 mM) to the test system prevented the PAF-specific elevation of [Ca^2+^]_c_, indicating that an influx of extracellular Ca^2+^ ions was probably responsible for the detected [Ca^2+^]_c_ increase (Fig 6B). Predictably, BAPTA had no effect on the Ca^2+^-response to PAF^D19S^ (Fig 6D).

### PAF and PAF^D19S^ act in a fungicidal, concentration and time dependent manner

**Fig 7:**
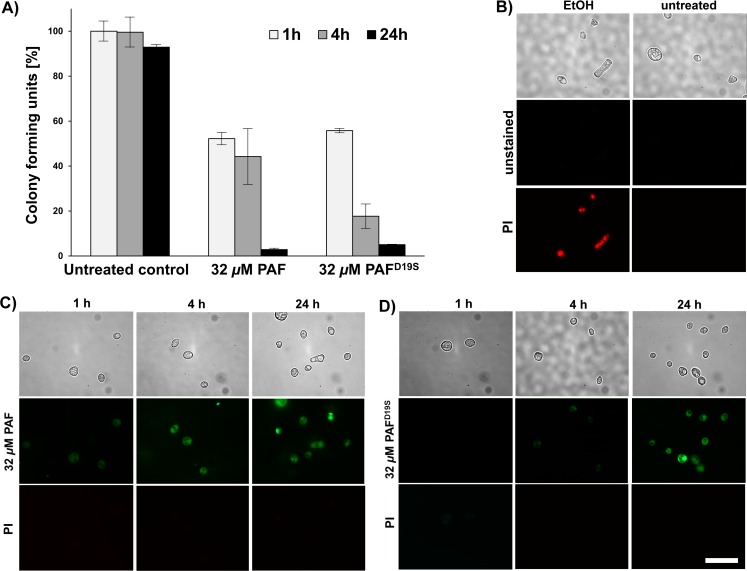
Viability of *N*. *crassa* conidia exposed to 32 μM PAF and PAF^D19S^. (***A***) Colony forming units were determined by counting the colonies emerging on agar plates after conidia had been treated for 1, 4 and 24 h, respectively, plated in appropriate dilutions and incubated for 24 h (untreated controls) or 48 h. Values are given in %-survival (untreated controls were set to be 100%) and represent the mean of three experiments ± standard errors. ***(B****)* Untreated conidia and conidia exposed to ethanol served as negative and positive staining controls, respectively. Conidia exposed to BODIPY-PAF (***C***) and BODIPY-PAF^D19S^ (***D***) for 1, 4 and 24 h were co-stained with PI. Scale bar = 30 μm.

To further dissect the functional difference between PAF and PAF^D19S^, both proteins were tested for fungistatic and fungicidal activities. To this end, *N*. *crassa* conidia were incubated with the antifungal proteins for 1, 4 and 24 h and appropriate dilutions were plated onto solid Vogel's agar to allow vital conidia to germinate and establish colonies. The survival rates were significantly reduced when exposing the conidia to high doses of PAF (32 μM) for 1 h (52 ± 2.6%, *p* = 0.00005) and 4 h (44 ± 12%, *p* = 0.03). This was further diminished after 24 h of incubation (3 ± 0.5%, *p* = 0.03) (Fig 7A). Instead, no significant reduction in the spore survival rate was detected with 0.13 μM PAF compared to the control (data not shown). This indicated that PAF acted in a fungistatic way at the concentration that corresponded to its MEC, but fungicidally at a 250-fold higher concentration in a time dependent way.

Instead, PAF^D19S^ acted fungicidal on conidia (survival rate: 56 ± 0.9%, *p* = 0.006) after 1 h of incubation at its MEC (32 μM). This effect was extenuated with prolonged incubation more rapidly as with PAF: the survival rate was reduced to 18 ± 5.3% (*p* = 0.004) after 4 h and to 5 ± 0.1% (*p* = 0.003) after 24 h of exposure (Fig 7A). No colony formation was detected with ethanol-treated controls.

Importantly, ungerminated *N*. *crassa* conidia exposed to the fungicidal concentration of 32 μM PAF or PAF^D19S^ showed no signs of cell death at any incubation time tested, as evaluated with PI staining (Fig 7B–7D). We therefore assume that PAF-induced cell death occurred exclusively in hyphae. To prove our hypothesis, *N*. *crassa* conidia were germinated in the presence of PAF and PAF^D19S^ for 6-30 h and then stained with PI. No increase in PI fluorescence was detected after 6 h of incubation with PAF (0.13 and 32 μM) compared to the untreated control. Instead, PI signals increased in hyphae after 24 h and reached a maximum after 30 h of exposure to 32 μM PAF (S10 Fig). Similar results were obtained with 32 μM PAF^D19S^ (data not shown).

## Discussion

Small, cationic and cysteine-rich proteins with antimicrobial activity are highly interesting bio-molecules for structural and functional research. Our study sheds important new light on the structural and mechanistic basis of the antifungal protein PAF, which may support the understanding of the function of other related antifungal proteins. A deep insight into the structure and function of these natural molecules is indispensable for future protein engineering and novel drug design, and more specifically for the rational design of improved PAF variants or PAF-derived synthetic peptides and their future applicability in new antifungal treatments.

### The structure, dynamics and thermal unfolding of PAF^D19S^ and their impact on antifungal function

We designed PAF^D19S^ as a part of our extensive mutation studies by which we wish to disclose unknown structure-function relations. Out of these attempts [7], PAF^D19S^ proved to be an extraordinary example where the mutation did not have a dramatic influence on the overall molecular structure, but caused a dramatic loss of antifungal activity. In our detailed NMR analyses we could show that the exchange of the solvent exposed and negatively charged aspartic acid 19 against a neutral serine perturbed internal molecular motions and the surface electrostatic distribution not only at the neighbourhood of the mutation, but at distant regions as well: for example in loop 3 (from Lys27 to Lys42) that involves the positively charged and mutation sensitive residues Lys34, Lys35 and Lys38 [7,31]. We propose that this is the consequence of the redistribution of charged side chains in absence of the negative charge of Asp19 in a “domino-like” manner: lysines near the mutated site are orienting towards other negative charges and this effect propagates via repulsion between lysines further on the surface. We suggest that this kind of “electrostatic allostery” is responsible for the observed changes in the surface and binding properties of the protein, as the lysines affected, together with Lys9 in loop 1 are highly conserved and located in near proximity in the folded protein, thus contributing to a cationic domain that is essential for full protein function [7,31]. A significant change in the charge of the protein surface may have severe impact on the interaction of PAF with its target molecule(s) and may explain the loss of antifungal function.

Another result raised our interest: the apparent difference between the thermal unfolding of PAF and PAF^D19S^. While ^15^N-relaxation data (which is sensitive to ps-ns molecular motions) revealed only slight local changes in the internal dynamics around the modified residue, the changes of the thermal unfolding parameters of the two protein forms extended into more regions. In our previous studies we demonstrated that PAF is a three-state folder and not a simple two-state folder: only a few residues could be fit with a two-state unfolding model, and this is attributed to the different timescale motions by which PAF interconverts between a few low-populated transitional states [8]. According to our present study PAF^D19S^ is rather a two-state folder and possesses less or less extended transition states than PAF. These NMR-invisible transition states may be essential for full antifungal action by enabling optimal target recognition, corroborating a putative role of intermediate states in the biological function of PAF [33]. Finally, our study provides for the first time a deeper understanding for the importance of an unconserved region, namely the Asn-Asp sequence in loop 2, for antifungal activity of PAF. To affirm our assumptions, we are currently generating PAF mutants with other amino acid substitutions in this loop to further dissect in detail its role in structure-function relation.

### PAF and PAF^D19S^ function is particularly Ca^2+^ ion sensitive

Current models for the mechanistic function of numerous antibacterial and antifungal acting cationic AMPs suggest that positively charged motifs on the protein surface are electrostatically attracted by the negatively charged phospholipid heads in the plasma membrane of microorganisms before the proteins insert into the membrane to evoke membrane perturbation and pore formation to ultimately trigger cell death [34,35]. This electrostatic interaction can be cation sensitive [36,37].

Indeed, the interaction of PAF and PAF^D19S^ with *N*. *crassa* was disturbed by elevated amounts of divalent cations, whereby the activity of both protein variants was most sensitive to Ca^2+^ ions. The specificity of the interference with divalent cations is further corroborated by the observation that monovalent cations like Na^+^ had no/little effect on the antifungal activity of PAF and PAF^D19S^. Whereas the role of Mg^2+^ ions in the activity of antifungal proteins awaits further investigations, the relevance of Ca^2+^ ions in the mechanistic function of PAF and other related antifungal proteins and peptides was studied in detail [23,28,31,38] and let assume that their role goes beyond the disruption of electrostatic interactions. We assume that Ca^2+^ ions may influence the PAF-response of the sensitive fungus in one or more of the following ways: (i) competition with PAF for binding to a Ca^2+^-sensitive interaction molecule, e.g. a receptor, pump, transporter or a binding protein, (ii) modulation of the distribution and/or activity of such binding molecules in the fungal cell membrane, or (iii) activation of resistance mechanisms, all of which ultimately impede PAF interaction.

### The killing mechanism of PAF in *N*. *crassa*

Cationic surface motifs of antifungal proteins and peptides that have no pore forming activity and are taken up into fungal cells to reach their site of action may determine not only the electrostatic attraction and interaction with the fungal cell but also regulate the intracellular transport triggering cell death [29]. Indeed, the uptake and the induction of a specific Ca^2+^ signature in susceptible fungal cells are prerequisites for the killing mechanism of PAF [23,30]. PAF and PAF^D19S^ were first attracted by the conidial cell envelope before they were internalized. This interaction *per se* was not detrimental to *N*. *crassa* as conidia showed no PI staining and still germinated in the presence of toxic concentrations of PAF and PAF^D19S^. Only following conidial germination and colony establishment the antifungal proteins fully acted on the actively growing hyphae in a concentration and time dependent manner. However, the primary killing mechanism of PAF and PAF^D19S^ was not the formation of pores in the plasma membrane because treated germlings were PI positive only after long incubation times. Such membrane damage could be a secondary effect after prolonged exposure to the antifungal proteins, for example as a result of increased oxidative stress induction in the cell [39]. A similar mode of action was proposed for antifungal plant defensins [40]. Instead, the increased vacuolation in PAF-treated hyphae suggested the onset of apoptotic events. Extensive hyphal vacuolation and other specific markers for apoptosis, e.g. elevated levels of reactive oxygen species, were previously identified in PAF-treated *Aspergillus nidulans* hyphae, which further proceeded to massive membrane damage [39]. We therefore favour the view that PAF induces the activation of specific signal transduction pathways that regulate growth inhibition and apoptosis, as proposed earlier [23,31,39,41].

Indeed, we could show that PAF^D19S^ failed to trigger a specific Ca^2+^ response even when applied at its MEC (32 μM) in contrast to PAF. In one of our previous studies we demonstrated that the PAF-specific rapid and sustained increase in [Ca^2+^]_c_ was inhibited when the test medium was supplemented with high Ca^2+^ concentrations [23,31]. This observation can be now directly linked to a significantly reduced interaction of PAF with fungal germlings in the presence of elevated amounts of extracellular Ca^2+^. Similarly, it has to be considered that PAF^D19S^ might not have reached a concentration on/in the fungal cells fast and/or high enough to induce a Ca^2+^ response even when tested in 0.2 x Vogel's medium with a low Ca^2+^ concentration (0.03 mM CaCl_2_). This assumption is supported by the observation of considerably reduced PAF^D19S^ binding and internalization in conidia and fungal cells compared to PAF. However, PAF^D19S^ acted fungicidal in a time dependent manner when applied at its MEC (32 μM) suggesting that fungal killing is mediated by other motifs that regulate mechanisms independently from the elevation of [Ca^2+^]_c_. Our observation parallels with recent studies on plant defensins: by using defensin fragments amino acid sequences were identified that exhibit distinct antifungal features of their parental proteins [28].

In summary, our study shows that the exchange of one critical negatively charged amino acid that resides in a non-conserved and dynamically flexible loop has severe impact on the protein surface charge and unfolding and affects the dynamics of distant protein motifs that are sensitive to surface modifications and essential for full PAF activity. This has to be considered in approaches that apply protein engineering for the development of natural bio-molecules with improved antifungal properties.

## Supporting Information

S1 Table Fungal strains used in this study. (PDF)

S2 Table Oligonucleotides used in this study. Mutation primers are in bold; mismatches for aa exchange are underlined.
(PDF)

S3 Table C_α_ and C_β_ chemical shifts (ppm) of PAF and PAF^D19S^. Missing resonances are due to low intensity NH and NH correlated peaks which is a consequence of H/D exchange of these peaks at pH = 6.0.
(PDF)

S1 Fig ESI-MS data of PAF^D19S^ (6.215 kDa), revealing the average isotopic pattern. Overviews spanning 4–8 kDa show the purity of the desired protein. MS results for PAF were published previously [7].
(TIF)

S2 Fig Structural differences between PAF and PAF^D19S^. Top: structural superposition of the NMR ensembles of PAF (green) and PAF^D19S^ (blue). Bottom: local RMSD for the two superimposed ensembles (purple line). Position of charged residues (cyan bars, pointing up: +1 charge, down: -1 charge) and local charge density (averaged over a 9-residue window, cyan line) is also shown. Figure prepared using MOLMOL and GnuPlot.
(TIF)

S3 Fig Comparison of lysine residues in PAF and PAF^D19S^. **(*A*)** Proximity table of lysine residues to negatively charged ones. For each Lys, the spatially closest Asp/Glu residue was identified in all 20 members of the NMR ensemble. Shading is proportional to the number the given contact was identified as closest. Atoms used for distance calculation: NZ for Lys, CD for Asp and CG for Glu residues. **(*B*)** Distribution of Lys dihedral angles in PAF (green) and PAF^D19S^ (blue). Either backbone or side-chain conformation is different for most Lys residues. Figure prepared using GnuPlot.
(TIF)

S4 Fig Selected examples of the CLEANEX experiment of PAF^D19S^ for measuring NH-H_2_O *k*_ex_ exchange rates. On the *x*-axis the variable mixing times in a series of CLEANEX experiments are shown as measured by the Bruker fhsqccxf3gpph pulse program. The measured peak volume integrals were referenced to the appropriate peak volumes of the fhsqcf3gpph fast HSQC spectrum. The normalized peak volumes were fit by the least square optimization routine of MATLAB, according to the theoretical equations given in [22]. Representative fits are shown for residues S19, A20, D23 and K30 with fitted *k*_ex_ rates of 19.2, 0.11, 1.51, and 0.19 s^-1^ exchange rates, respectively. The experiments work best around *k*_ex_ ≈1 s^-1^, because in that case sufficient magnetization is detected in the initial period.
(TIF)

S5 Fig ^15^N-^1^H *S*^2^order parameters (*y* axis) of PAF (green) and of PAF^D19S^ (blue) as a function of residue number (*x* axis). Order parameters were obtained from the Lipari-Szabó analysis of ^15^N T_1_, T_2_ and ^15^N-{^1^H} NOE experimental relaxation data, and processed using Bruker Protein Dynamics Center 2.2.4. package, M2 model. PAF relaxation data were used from Batta et al. [7], supplementary material. The averaged *S*^2^ parameters are: *S*^2^ = 0.81 ± 0.05 (for PAF) and *S*^2^ = 0.78 ± 0.05 (for PAF^D19S^). Standard deviation error limits as obtained by Monte-Carlo analysis (1000 steps) are shown.
(TIF)

S6 Fig ^15^N-CEST intensity profile of D53 residue in PAF^D19S^. ^15^N saturation is shown as the function of ^15^N-offset during the CEST experiment. The bigger peak represents the major conformation, and the small peak represents low populated protein fractions at specific ^15^N chemical shifts. This minor conformation is in slow exchange with the visible native conformer. CEST experiment proves that these low-populated conformations are present in PAF and PAF^D19S^ as well.
(TIF)

S7 Fig Errors of fit by the two-state thermal unfolding model for PAF (green) and PAF^D19S^ (blue). Dotted red line indicates the arbitrary 6% error limit level for two state folder residues.
(TIF)

S8 Fig Localization of BODIPY-PAF upon contact with 6 h-old *N*. *crassa* germlings. Five minutes after exposure to 4 μM PAF signal intensity was highest at outer cell layers of conidia. Panels represent blue fluorescence of calcofluor white (CFW) cell wall stain **(*A*)**, pre-treatment with 50 μg/mL CFW for 15 min before addition of BODIPY-PAF, green fluorescence of BODIPY-PAF **(*B*)**, merged fluorescence images **(*C*)** and bright-field image **(*D*)**. Scale bar = 5 μm.
(TIF)

S9 Fig Localization of BODIPY-PAF in 6 h-old *N*. *crassa* and *A*. *terreus* germlings. Specific fluorescent signals are visible in *N*. *crassa* after 15, 30 and 60 min of incubation with 0.8 μM antifungal protein, whereas no signals could be detected in the PAF-resistant control strain *A*. *terreus*, exposed to 32 μM labelled protein for 60 min. Scale bars = 30 μm (overviews) & 5 μm (insets).
(TIF)

S10 Fig The viability of germlings after exposure to 0.13 or 32 μM PAF for different time periods. Germlings treated with 70% ethanol (EtOH) for 15 min were used as positive PI staining controls, untreated germlings served as negative controls. Dead cells with compromised plasma membrane show strong intracellular red fluorescence. Light microscopy images are overlaid with the fluorescent images. Scale bar = 50 μm.
(TIF)
