# Supplementary material for: D19S Mutation of the Cationic, Cysteine-Rich Protein PAF: Novel Insights into Its Structural Dynamics, Thermal Unfolding and Antifungal Function
Source: PLoS One. 2017 Jan 10;12(1):e0169920. doi: 10.1371/journal.pone.0169920 (PMC5224997; doi:10.1371/journal.pone.0169920)
Supplement: S1 Table — (PDF) [file pone.0169920.s001.pdf]

S1 Table. Fungal strains used in this study.

Strain	Genotype	Reference
N. crassa 74-OR23-1A	wild-type mat A	FGSC 2489
N. crassa-AEQ	*hygR aeqS*	Zelter et al. 2004 [27]
P. chrysogenum Q176	wild-type	ATCC 10002
P. chrysogenum Δ*paf*	Δ*paf*:*nat1*	Hegedüs et al. 2011 [14]
P. chrysogenum C3	Δ*paf*:*nat1, paf*$^{+}$, *ptrA*$^{+}$	this study
P. chrysogenum PAF^{D19S}	Δ*paf*:*nat1, paf*^{D19S}, *ptrA*$^{+}$	this study
