# Supplementary material for: D19S Mutation of the Cationic, Cysteine-Rich Protein PAF: Novel Insights into Its Structural Dynamics, Thermal Unfolding and Antifungal Function
Source: PLoS One. 2017 Jan 10;12(1):e0169920. doi: 10.1371/journal.pone.0169920 (PMC5224997; doi:10.1371/journal.pone.0169920)
Supplement: S2 Table — Mutation primers are in bold; mismatches for aa exchange are underlined. (PDF) [file pone.0169920.s002.pdf]

Table S2. Oligonucleotides used in this study. Mutation primers are in bold; mismatches for aa exchange are underlined.

Name	Sequence 5'-3'
M13	GTAAAACGACGGCCAGTGAG
T7var	TACGACTCACTATAGGGCG
opaf11	CACTCCCCTCATACTTCATG
opaf12	CTTCTCTGACTGAAAGTACC
**opafD19Sfw**	**AAATACAAGAAC<u>TCC</u>GCTGGAAAGGAC**
**opafD19Srev**	**CCTTTCCAGC<u>GGA</u>GTTCTTGTATTTAC**
