# Supplementary material for: D19S Mutation of the Cationic, Cysteine-Rich Protein PAF: Novel Insights into Its Structural Dynamics, Thermal Unfolding and Antifungal Function
Source: PLoS One. 2017 Jan 10;12(1):e0169920. doi: 10.1371/journal.pone.0169920 (PMC5224997; doi:10.1371/journal.pone.0169920)
Supplement: S3 Table — Missing resonances are due to low intensity NH and NH correlated peaks which is a consequence of H/D exchange of these peaks at pH = 6.0. (PDF) [file pone.0169920.s003.pdf]

S3 Table. C_α and C_β chemical shifts (ppm) of PAF and PAF^{D19S}. Missing resonances are due to low intensity NH and NH correlated peaks which is a consequence of H/D exchange of these peaks at pH = 6.0.

Residue No.	Amino acid	PAF		PAF^{D19S}	
		C_α	C_β	C_α	C_β
1	ALA	49.162	17.107		
2	LYS	52.189	31.708		
3	TYR	53.741	39.69	53.764	39.643
4	THR	58.808	67.837	58.821	67.74
5	GLY	42.066		42.102	
6	LYS	51.644	34.997	51.603	34.991
7	CYS	50.41	42.645	50.398	42.613
8	THR	57.291	68.608	57.302	68.384
9	LYS	57.196	31.079	57.189	31.086
10	SER	59.045			59.099
11	LYS	53.362	30.55	53.31	30.598
12	ASN	50.874	34.483	50.901	34.525
13	GLU	52.029	33.773	52.174	32.517
14	CYS	51.114	39.542	51.127	39.486
15	LYS	51.435	31.472	51.708	31.663
16	TYR	52.093	38.479	52.146	38.551
17	LYS	51.694	30.894	51.84	30.133
18	ASN	48.158	35.143	48.311	35.274
19	ASP/SER	54.265	37.359		60.567
20	ALA	48.765	16.013	48.861	16.183
21	GLY	42.827		42.798	
22	LYS	51.436	30.576	51.68	30.128
23	ASP	52.973	38.123	53.037	38.074
24	THR	59.223	67.985	59.189	67.965
25	PHE	53.597	39.245	53.644	39.241
26	ILE	56.844	39.475	57.036	39.506
27	LYS	55.166	29.58	55.201	29.593
28	CYS	52.541	38.587	53.957	38.484
30	LYS	53.497	30.09	53.539	30.131
31	PHE	54.044	37.643	54.085	37.631
32	ASP	54.793	37.892		37.931
33	ASN	51.362	34.754	51.397	34.822
34	LYS	52.148	32.813	52.559	32.796
35	LYS	52.155	30.775	52.388	30.786
36	CYS	53.356	38.703		38.731
37	THR	60.478	68.235	60.573	
38	LYS	53.865	32.499	53.899	32.522
39	ASP	54.033	37.454	52.668	37.403
40	ASN	52.975	34.18	53.02	34.177
41	ASN	49.368	36.398		36.351
42	LYS	54.55	30.627	54.574	30.609

43	CYS	50.891		51.063	47.219
44	THR	57.308	69.607	57.375	69.497
45	VAL	58.069	33.384	58.057	33.248
46	ASP	49.255	40.051	49.43	40.094
47	THR	60.559	66.582	60.713	66.315
48	TYR	57.619	35.817	57.59	35.765
49	ASN	49.231	34.484	49.245	35.79
50	ASN	51.663	34.202	51.695	34.292
51	ALA	49.603	17.688	49.733	17.643
52	VAL	57.894	32.369	57.939	32.397
53	ASP	50.331	40.762	50.353	40.868
54	CYS	51.047	41.549	53.504	41.804
55	ASP	52.776	39.595	52.798	39.599
