# Supplementary figures and images for: D19S Mutation of the Cationic, Cysteine-Rich Protein PAF: Novel Insights into Its Structural Dynamics, Thermal Unfolding and Antifungal Function
Source: PLoS One. 2017 Jan 10;12(1):e0169920. doi: 10.1371/journal.pone.0169920 (PMC5224997; doi:10.1371/journal.pone.0169920)

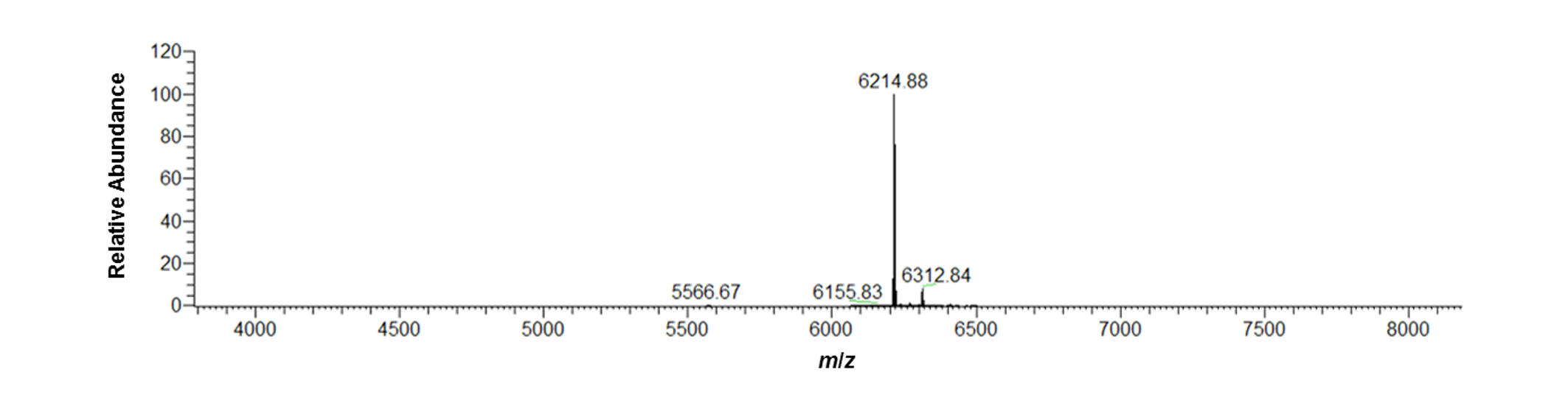

Supplement: S1 Fig — Overviews spanning 4–8 kDa show the purity of the desired protein. MS results for PAF were published previously [7]. (TIF) [file pone.0169920.s004.tif]

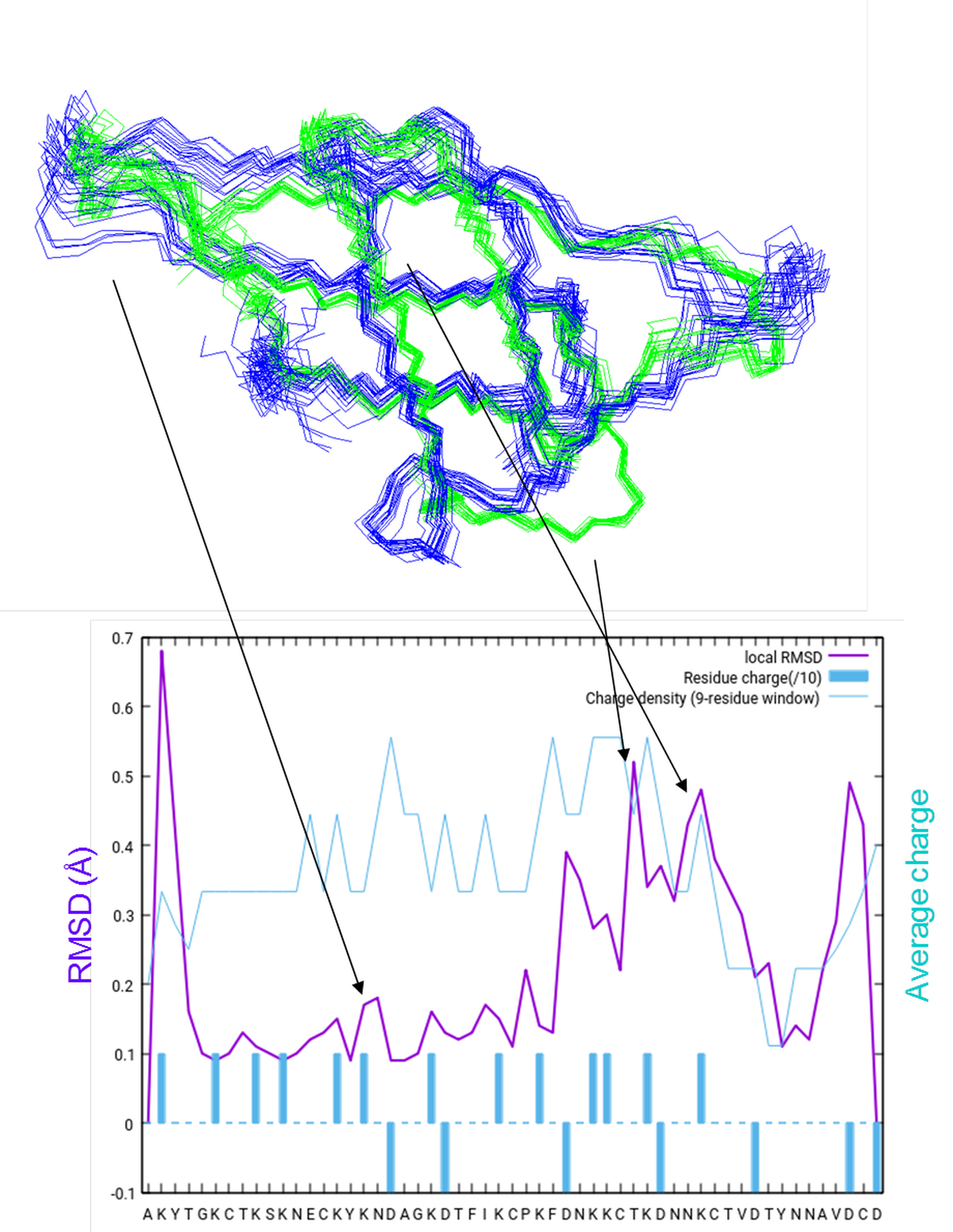

Supplement: S2 Fig — Top: structural superposition of the NMR ensembles of PAF (green) and PAFD19S (blue). Bottom: local RMSD for the two superimposed ensembles (purple line). Position of charged residues (cyan bars, pointing up: +1 charge, down: -1 charge) and local charge density (averaged over a 9-residue window, cyan line) is also shown. Figure prepared using MOLMOL and GnuPlot. (TIF) [file pone.0169920.s005.tif]

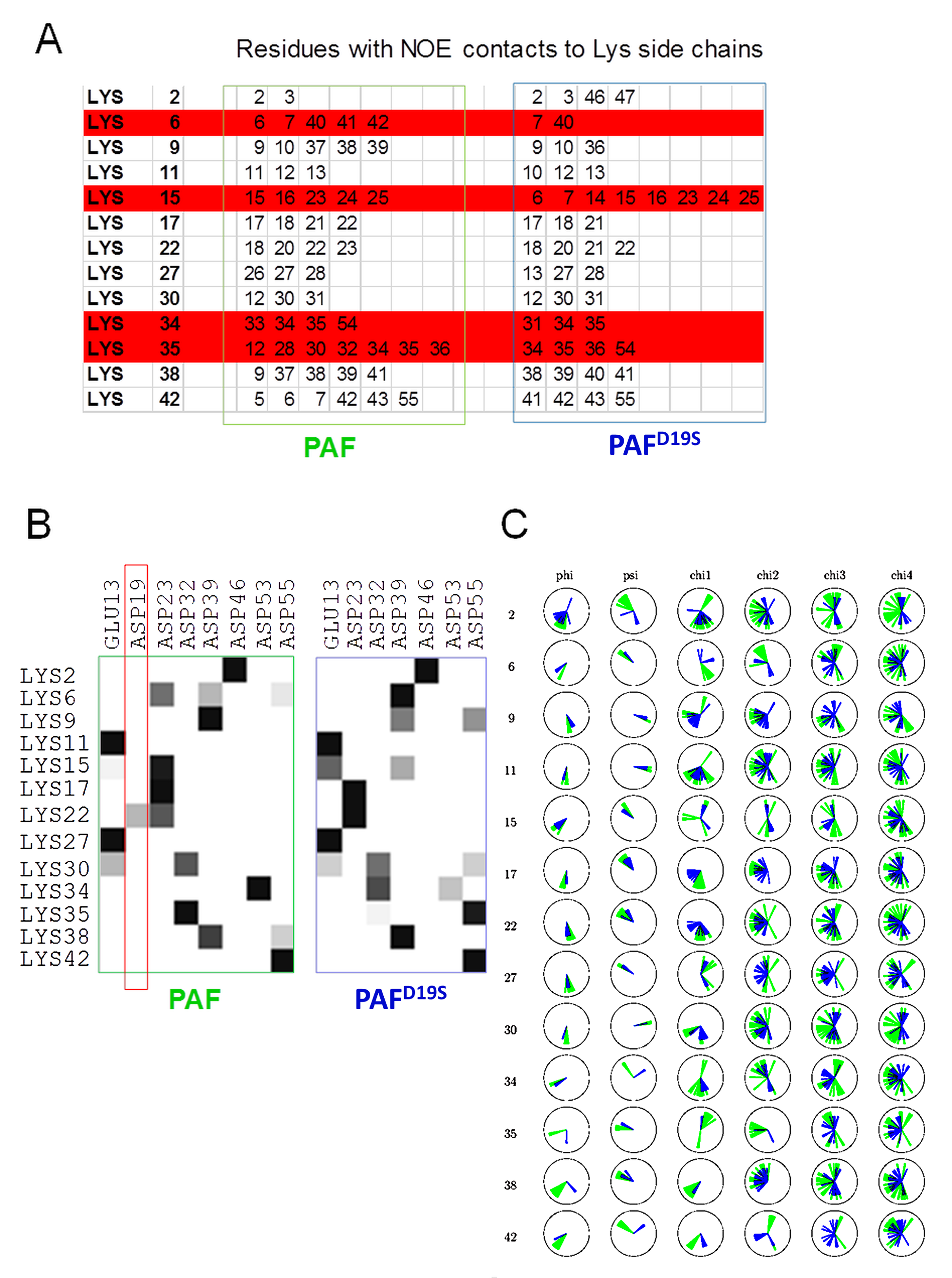

Supplement: S3 Fig — (A) Proximity table of lysine residues to negatively charged ones. For each Lys, the spatially closest Asp/Glu residue was identified in all 20 members of the NMR ensemble. Shading is proportional to the number the given contact was identified as closest. Atoms used for distance calculation: NZ for Lys, CD for Asp and CG for Glu residues. (B) Distribution of Lys dihedral angles in PAF (green) and PAFD19S (blue). Either backbone or side-chain conformation is different for most Lys residues. Figure prepared using GnuPlot. (TIF) [file pone.0169920.s006.tif]

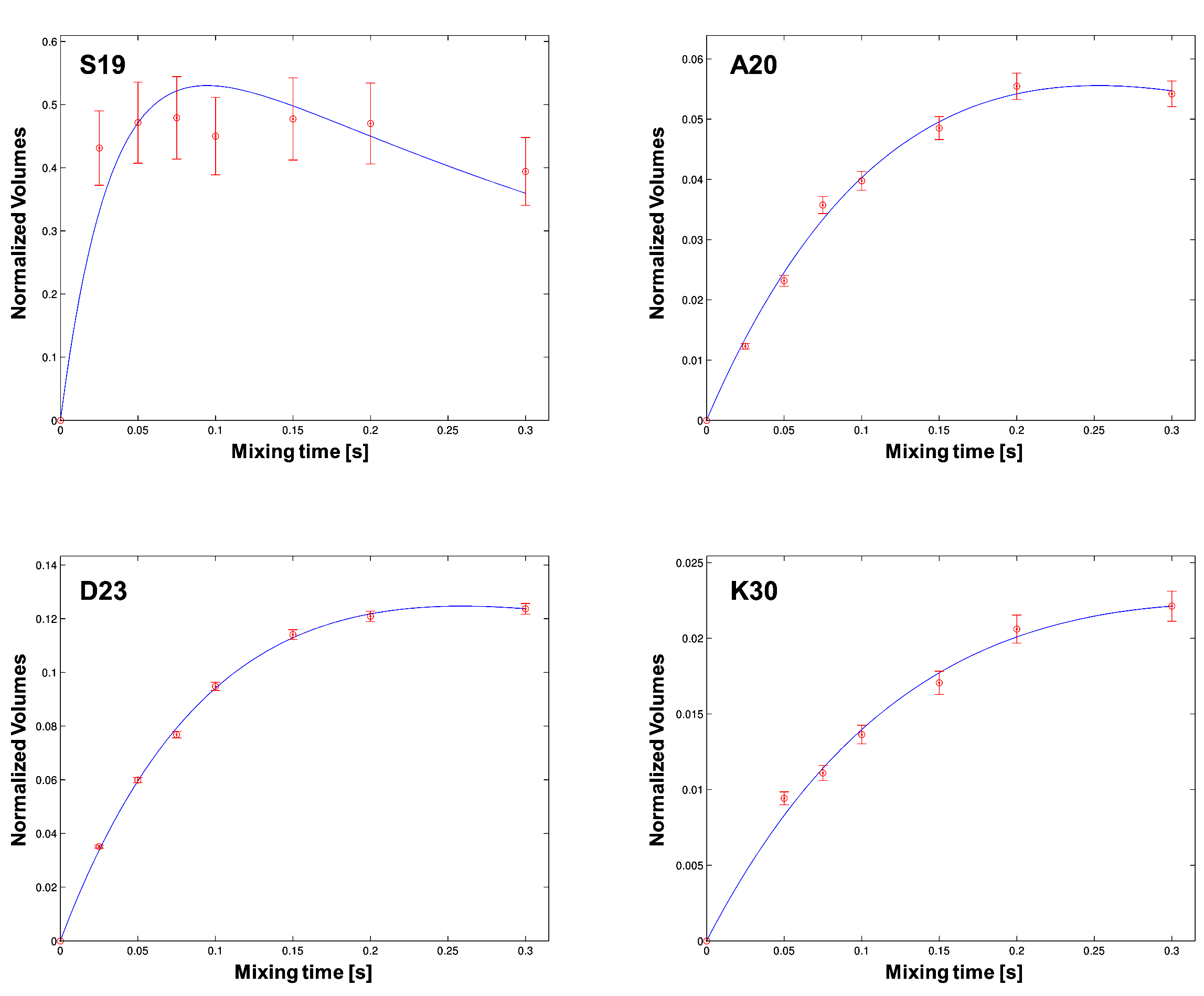

Supplement: S4 Fig — On the x-axis the variable mixing times in a series of CLEANEX experiments are shown as measured by the Bruker fhsqccxf3gpph pulse program. The measured peak volume integrals were referenced to the appropriate peak volumes of the fhsqcf3gpph fast HSQC spectrum. The normalized peak volumes were fit by the least square optimization routine of MATLAB, according to the theoretical equations given in [22]. Representative fits are shown for residues S19, A20, D23 and K30 with fitted kex rates of 19.2, 0.11, 1.51, and 0.19 s-1 exchange rates, respectively. The experiments work best around kex ≈1 s-1, because in that case sufficient magnetization is detected in the initial period. (TIF) [file pone.0169920.s007.tif]

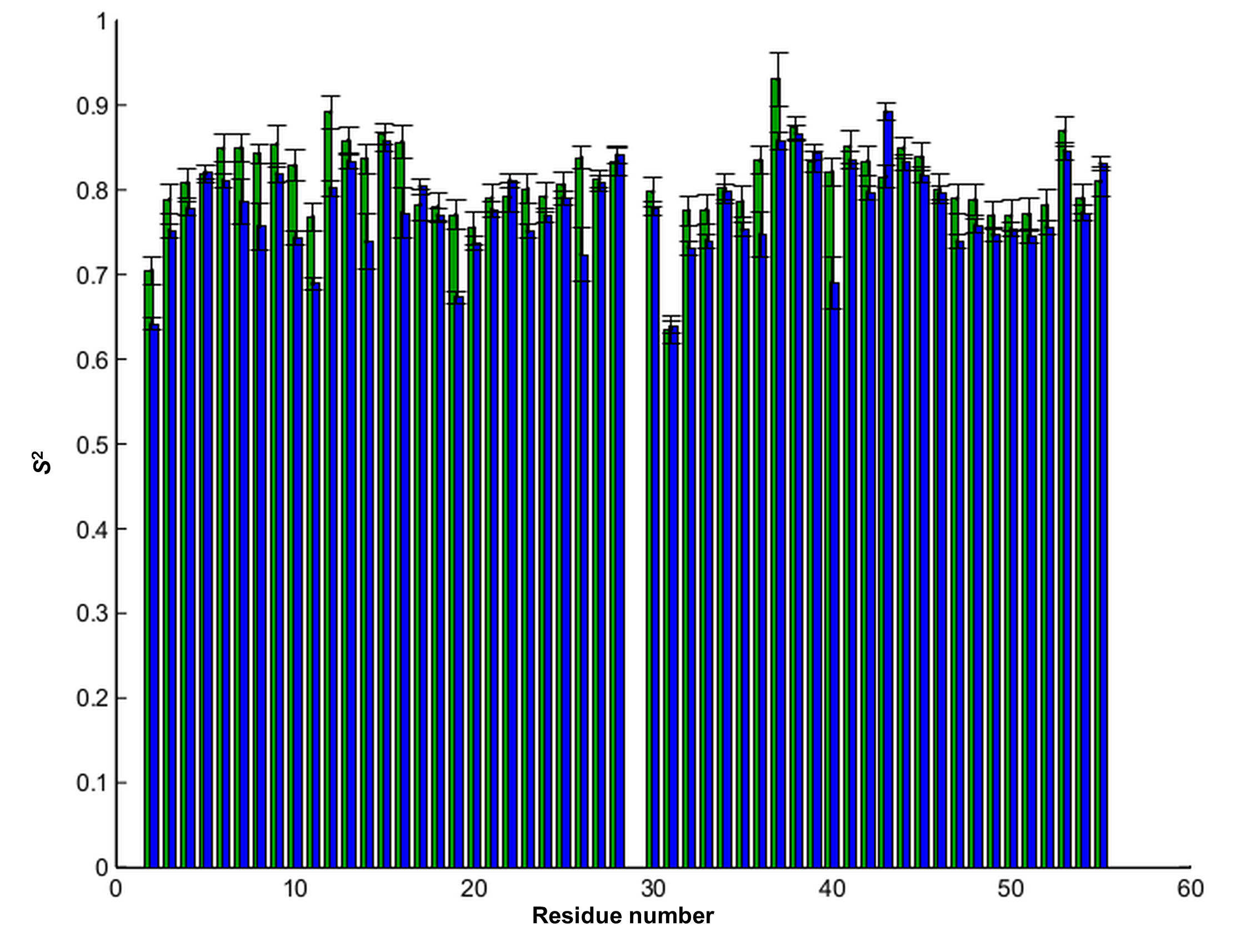

Supplement: S5 Fig — Order parameters were obtained from the Lipari-Szabó analysis of 15N T1, T2 and 15N-{1H} NOE experimental relaxation data, and processed using Bruker Protein Dynamics Center 2.2.4. package, M2 model. PAF relaxation data were used from Batta et al. [7], supplementary material. The averaged S2 parameters are: S2 = 0.81 ± 0.05 (for PAF) and S2 = 0.78 ± 0.05 (for PAFD19S). Standard deviation error limits as obtained by Monte-Carlo analysis (1000 steps) are shown. (TIF) [file pone.0169920.s008.tif]

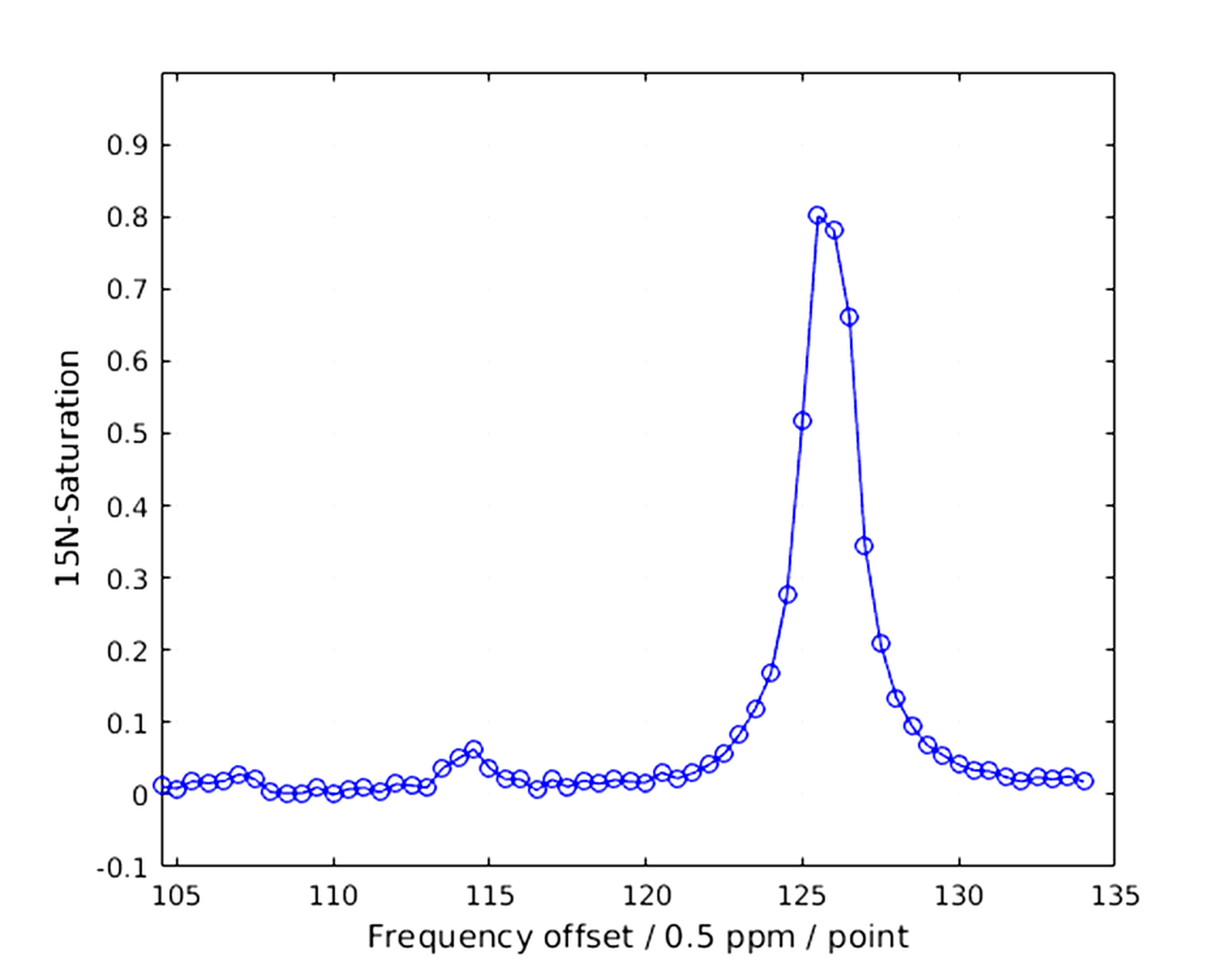

Supplement: S6 Fig — 15N saturation is shown as the function of 15N-offset during the CEST experiment. The bigger peak represents the major conformation, and the small peak represents low populated protein fractions at specific 15N chemical shifts. This minor conformation is in slow exchange with the visible native conformer. CEST experiment proves that these low-populated conformations are present in PAF and PAFD19S as well. (TIF) [file pone.0169920.s009.tif]

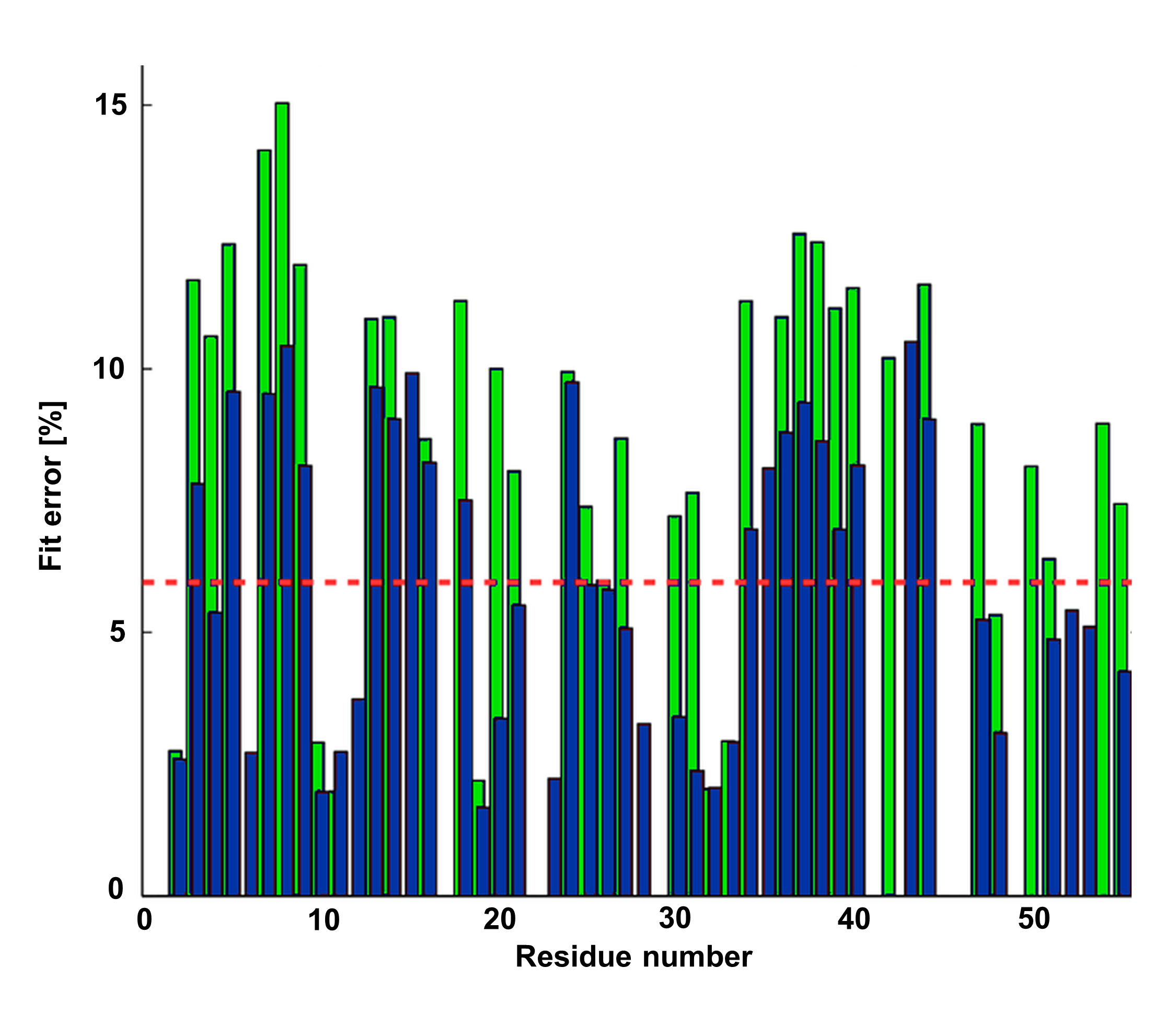

Supplement: S7 Fig — Dotted red line indicates the arbitrary 6% error limit level for two state folder residues. (TIF) [file pone.0169920.s010.tif]

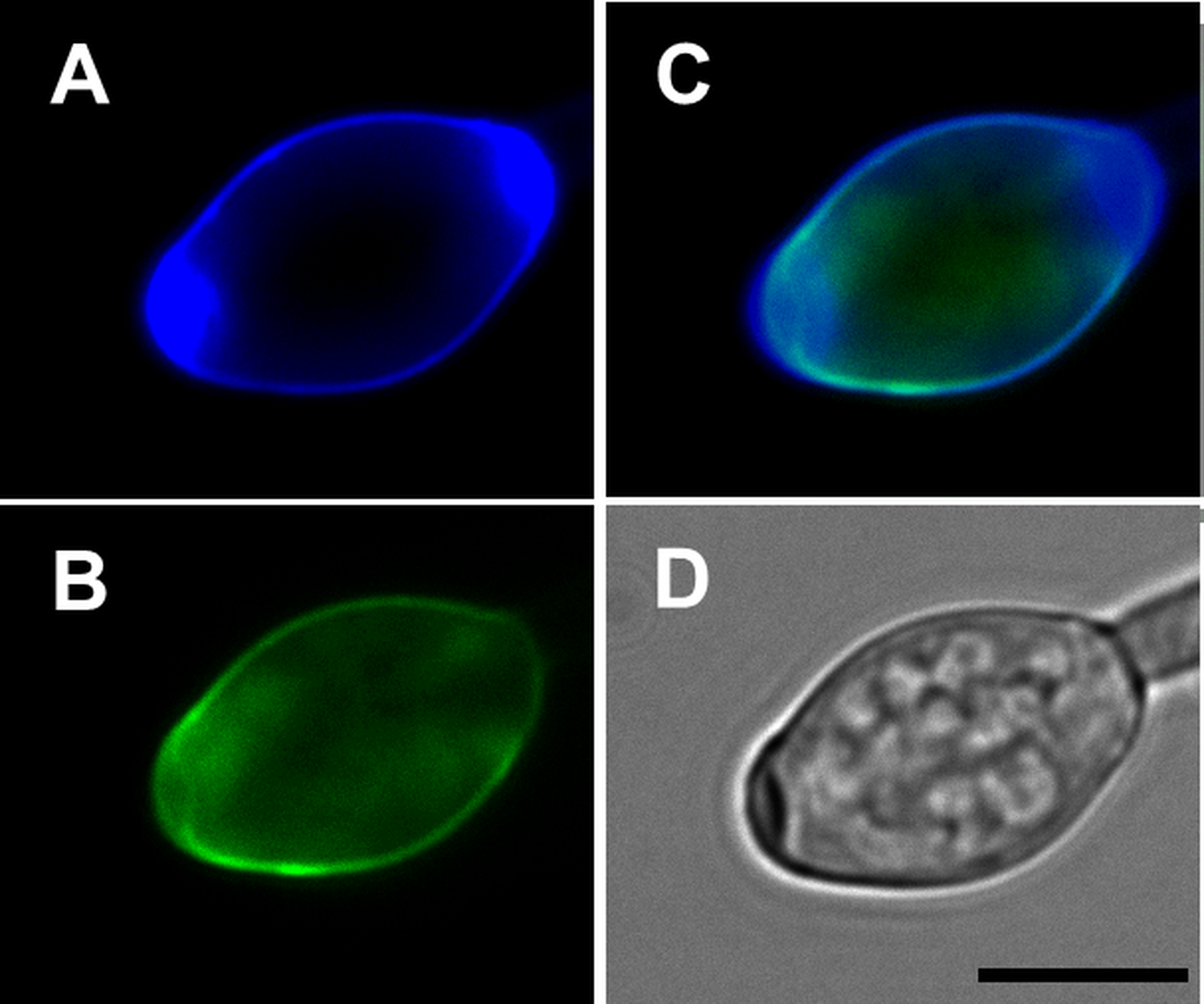

Supplement: S8 Fig — Five minutes after exposure to 4 μM PAF signal intensity was highest at outer cell layers of conidia. Panels represent blue fluorescence of calcofluor white (CFW) cell wall stain (A), pre-treatment with 50 μg/mL CFW for 15 min before addition of BODIPY-PAF, green fluorescence of BODIPY-PAF (B), merged fluorescence images (C) and bright-field image (D). Scale bar = 5 μm. (TIF) [file pone.0169920.s011.tif]

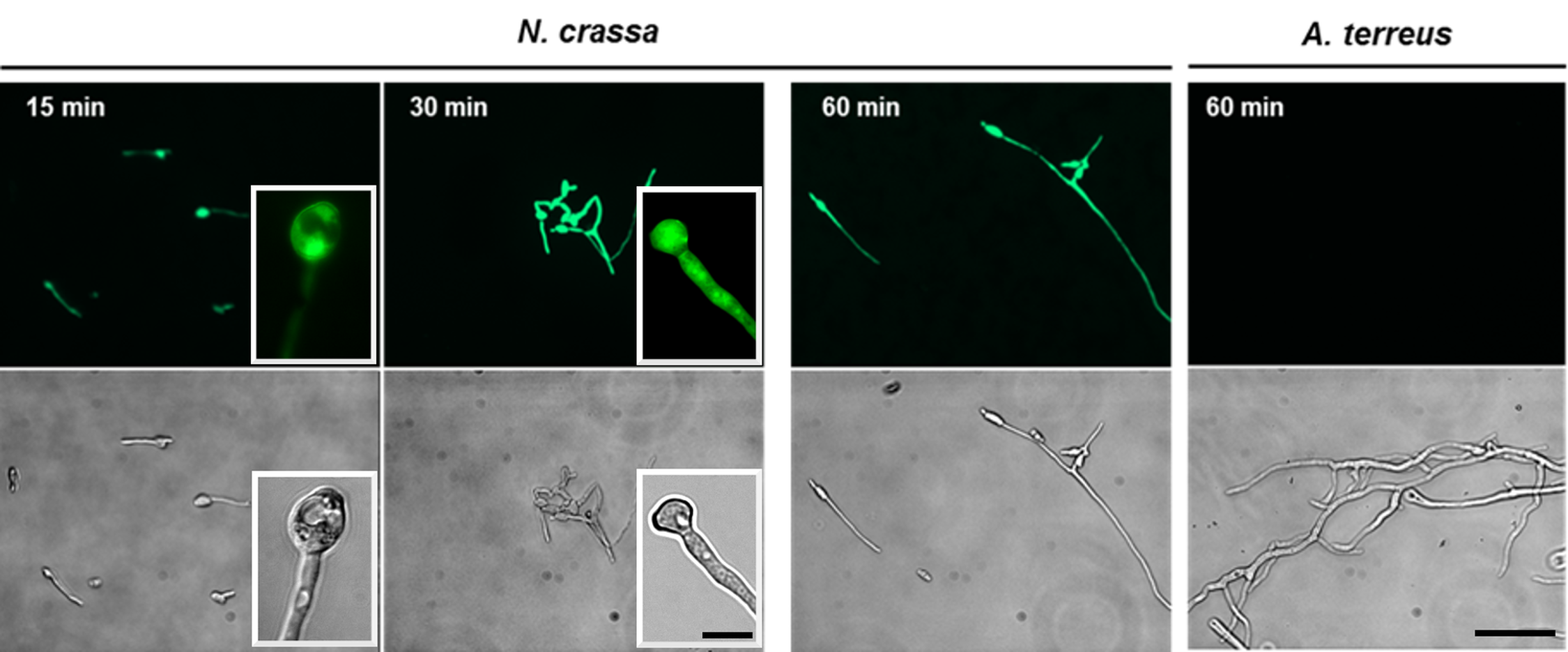

Supplement: S9 Fig — Specific fluorescent signals are visible in N. crassa after 15, 30 and 60 min of incubation with 0.8 μM antifungal protein, whereas no signals could be detected in the PAF-resistant control strain A. terreus, exposed to 32 μM labelled protein for 60 min. Scale bars = 30 μm (overviews) & 5 μm (insets). (TIF) [file pone.0169920.s012.tif]

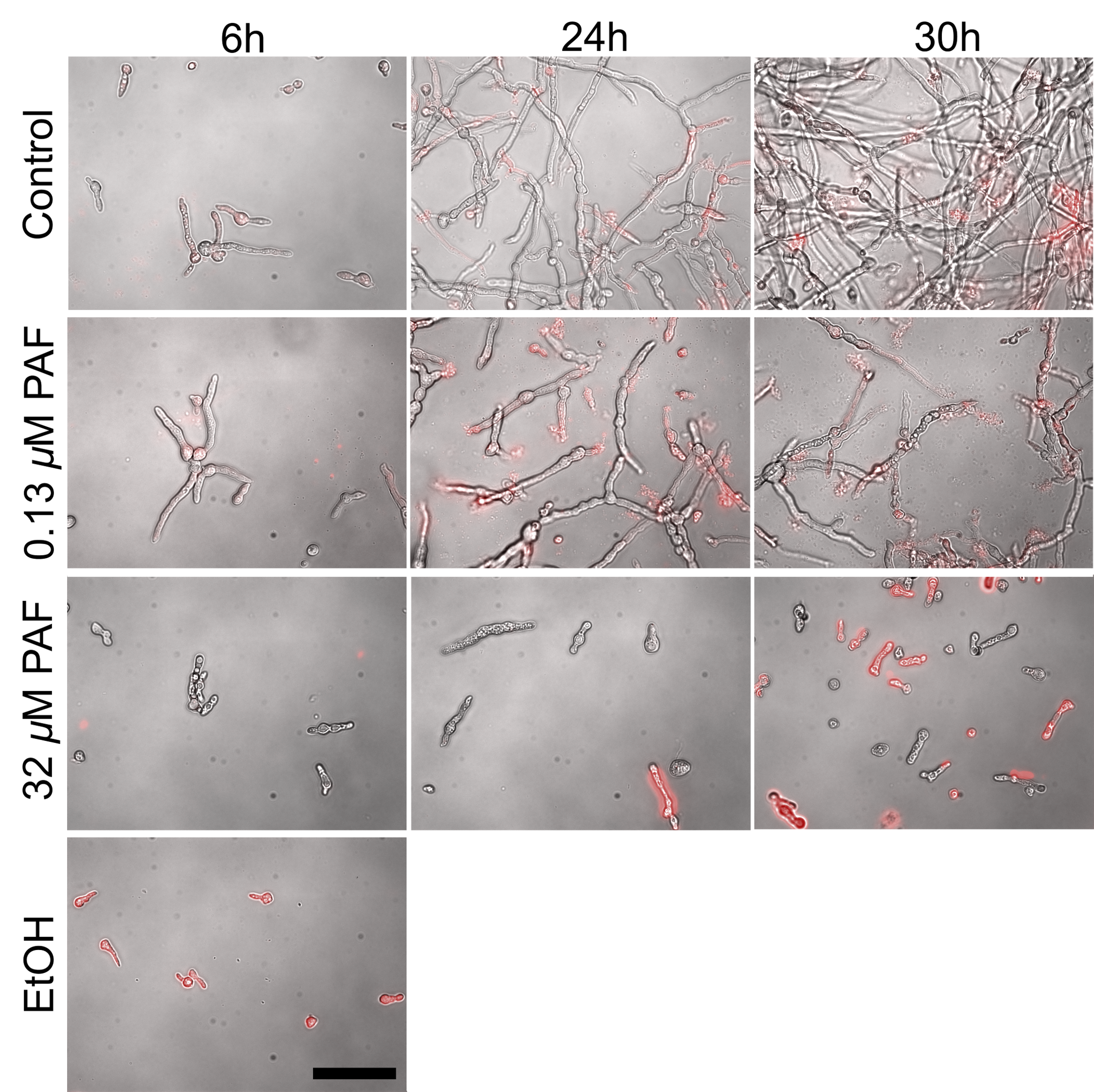

Supplement: S10 Fig — Germlings treated with 70% ethanol (EtOH) for 15 min were used as positive PI staining controls, untreated germlings served as negative controls. Dead cells with compromised plasma membrane show strong intracellular red fluorescence. Light microscopy images are overlaid with the fluorescent images. Scale bar = 50 μm. (TIF) [file pone.0169920.s013.tif]
